# Enzyme-Assisted Ultrasonic Extraction of Flavonoids from *Pinus koraiensis* Needle Litterfall: Process Optimization, Component Identification, and In Vitro Bioactivity Evaluation

**DOI:** 10.3390/antiox15060712

**Published:** 2026-06-03

**Authors:** Weiwei Liang, Le Ouyang, Chun Bian, Yuxin Shan, Xiufang Xia

**Affiliations:** 1 College of Food Science, Northeast Agricultural University, Harbin 150030, China; lww88@hrbu.edu.cn; 2 College of Food Engineering, Harbin University, Harbin 150086, China; oyl2022@hrbu.edu.cn (L.O.); bianchun1980@126.com (C.B.); 15776093541@163.com (Y.S.)

**Keywords:** *Pinus koraiensis* needle, enzyme-assisted ultrasonic extraction, flavonoids, response surface methodology, DPPH radical and ABTS cation radical scavenging activity, α-amylase- and α-glucosidase-inhibitory activities

## Abstract

Flavonoids from *Pinus koraiensis* needle (PN) litterfall were efficiently recovered using an enzyme-assisted ultrasonic extraction (EAU) method optimized via response surface methodology (RSM). The optimal conditions (enzyme dosage 1.7%, ethanol concentration 70%, ultrasonic time 21 min, cellulase–pectinase ratio 1:3, liquid–solid ratio 40:1, enzymatic hydrolysis at 42.5 °C for 1 h, ultrasonic extraction at 50 °C and 150 W) yielded a total flavonoid content (*TFC*) of 17.08 mg rutin/g, which was significantly higher than that obtained via conventional extraction (CE). Scanning electron microscopy (SEM) confirmed that the treatment disrupted the cell wall, promoting flavonoid release. Ultra-performance liquid chromatography coupled with triple-quadrupole time-of-flight mass spectrometry (UPLC-Triple-TOF/MS) identified 60 flavonoids in the purified extract obtained under the optimal EAU conditions (OT group), including quercitrin, tiliroside, taxifolin, and procyanidin B2. Fourier-transform infrared spectroscopy (FTIR) and differential scanning calorimetry (DSC) showed higher crystallinity but slightly reduced thermal stability for OT flavonoids. Notably, compared with the purified flavonoids obtained by CE (CK1 group), the OT group achieved a higher *TFC* and exhibited significantly better in vitro antioxidant activity (DPPH IC_50_ = 71.82 μg/mL; ABTS IC_50_ = 28.93 μg/mL) and in vitro carbohydrate-digesting-enzyme-inhibitory activity (α-glucosidase (α-GLU) IC_50_ = 79.52 μg/mL; α-amylase (α-AMY) IC_50_ = 793.9 μg/mL), with α-AMY inhibition being approximately 8.2-fold higher. These findings suggest that enzyme-assisted ultrasonic extraction is an efficient and reliable method for recovering flavonoids from PN and may provide a theoretical reference for the development and utilization of these flavonoids.

## 1. Introduction

Flavonoids are a class of secondary plant metabolites characterized by a 2-phenylchromone structure and serve as important bioactive constituents in numerous plants [1]; they can be classified into eight major subclasses—flavones, flavonols, isoflavones, chalcones, dihydrochalcones, flavanones, flavanols, and anthocyanins—with representative compounds including quercetin, kaempferol, and apigenin [2]. Flavonoids exhibit a broad spectrum of biological activities, such as anti-inflammatory, antioxidant, antiviral, and antimicrobial effects [3,4]; they also show potential in preventing chronic diseases, including tumors, cardiovascular disorders, and neurodegenerative conditions [5,6], making them promising candidates for pharmaceuticals and nutraceuticals aimed at managing obesity, diabetes, and related metabolic syndromes. To date, the flavonoid profiles and functional properties of many plant species have been extensively analyzed and characterized [7,8,9].

*Pinus koraiensis* Siebold & Zucc. is a species of the genus *Pinus* (family Pinaceae); it typically grows on shady or semi-shady slopes at high altitudes or on moist, fertile mid-mountain slopes. The species is predominantly distributed in cold, humid, brown forest soil zones, mainly from the Lesser Khingan Mountains to the Changbai Mountains in northeastern China, as well as in parts of Russia, Japan, and the Korean Peninsula [10]. Various parts of the tree, such as its bark, cones, and needles, have been utilized in traditional medicine. *Pinus koraiensis* exhibits strong cold resistance, and its wood is soft, rich in resin, and highly decay-resistant, making it valuable for multiple applications. The needles and cones are particularly notable as sources of antioxidant bioactive compounds [11,12]. Pine needles, also known as pine leaves or pine hairs, are important vegetative organs of *Pinus* species, characterized by rapid growth, large natural reserves, wide distribution, and year-round harvestability [13]. For a long time, their potential value has been significantly underestimated. In traditional forestry production, large quantities of pine needle resources are typically burned or left to decompose naturally as forest waste. This highly flammable material is a major cause of forest fires, which not only cause severe damage to plant and animal communities and forest lands but also emit greenhouse gases into the atmosphere, resulting in substantial resource waste [14]. In fact, pine needles possess high research and application value. Ancient Chinese medical classics, such as the *Compendium of Bencao Gangmu* and the *Huiyue Yijing*, document that pine needles can replenish vital energy, promote longevity, and relieve thirst. Modern studies have shown that pine needle tissues contain a variety of bioactive components [15,16,17], including abundant cellulose, essential oils, and flavonoids, which exhibit anti-inflammatory, antioxidant, anti-fatigue, anti-aging, and antibacterial effects. Thus, pine needles represent a natural source of health-promoting nutritional supplements and pharmaceutical ingredients [18]. Given the current low utilization rate of pine needle resources, research into the extraction and application of bioactive components from pine needles is urgently needed.

Shi et al. [19] used a 40% ethanol heat-reflux extraction method combined with HPD722 macroporous adsorption resin to purify total flavonoids from *Cedrus deodara* pine needles, achieving a flavonoid purity of 54.28%. Several bioactive flavonoid monomers were identified in the purified product, including myricetin (1.89 mg/g), quercetin (2.01 mg/g), kaempferol (2.94 mg/g), and isorhamnetin (1.22 mg/g). The total flavonoids significantly inhibited the proliferation of HepG2 cells, even at low doses, confirming their in vitro anticancer potential by modulating the cell cycle and apoptosis. Kurtiš et al. [20] compared three extraction methods (80% methanol conventional maceration, ultrasound-assisted extraction, and digestion) for extracting flavonoids from the needles of five pine species (*Pinus sylvestris*, *Pinus nigra*, *Pinus heldreichii*, *Pinus halepensis*, and *Pinus pinea*) from Montenegro. The results showed that the *TFC* varied depending on both species and extraction method. The highest content (1.213 mg QE/g) was obtained via digestion from *Pinus sylvestris*. Ultrasound-assisted extraction yielded 1.074 mg QE/g for both *Pinus heldreichii* and *Pinus halepensis*. The extracts exhibited different antioxidant mechanisms: generally, DPPH activity showed little variation, while FRAP activity varied significantly. Regarding antibacterial activity, all extracts were more effective against Gram-positive bacteria (e.g., *Bacillus spizizenii* and *Staphylococcus aureus*) than against Gram-negative strains. Toxicity assays revealed no significant cytotoxicity in any of the extracts at the tested concentrations. This study provides a scientific basis for the valorization of Montenegrin pine needle resources as natural antioxidants and antibacterial agents. Geana et al. [21] investigated microwave-assisted extraction (using methanol and ethanol) of bioactive compounds from the bark and needles of softwood species (*Picea abies* L., H. Karst., and *Abies alba* Mill.), followed by enrichment via liquid–liquid extraction. The chemical analysis revealed that needle extracts were rich in various flavonoids, including (+)- catechin, epicatechin, kaempferol and its glycosides, myricetin, and rutin. The extracts exhibited remarkable antioxidant activity in DPPH assays, with a dose-dependent effect. In wound-healing assays, the extracts promoted fibroblast migration and proliferation, demonstrating their potential to accelerate wound healing. This study highlights softwood needle extracts as promising natural sources of antioxidants and promoters of wound healing. Tbatou et al. [22] extracted flavonoids from the needles of *Pinus pinaster* Aiton (PPN) and *Pinus halepensis* Mill. (PAN) using 70% ethanol and investigated their chemical composition, as well as their antioxidant, analgesic, and wound-healing activities. The *TFC* values were 109.44 ± 0.62 and 111.64 ± 0.62 mg QE/g DM for PPN and PAN, respectively. Several compounds were identified, including myricetin-3-O-glucoside (*m*/*z* 479.28), quercetin-3-O-glucoside (*m*/*z* 463.33), and p-O-Coumaroylquinic acid and its derivatives (*m*/*z* 337.21); both extracts showed significant antioxidant activity. In analgesic activity tests, the PAN extract (300 mg/kg) reduced pain by 72.3%, with a central analgesic effect comparable to that of the reference drug. Both extracts accelerated wound contraction, achieving complete closure within 21 days, indicating good tissue regeneration potential. In summary, flavonoids from pine needles exhibit multiple bioactivities, the intensity of which is closely related to the pine species, extraction and purification methods, solvent system, and target compounds. The systematic optimization of extraction processes and comprehensive chemical characterization are therefore essential for unlocking the functional potential of pine needle resources.

Optimization is a technical approach that systematically seeks the optimal process based on predefined quality criteria and constraints, with the core objective of improving the yield of target products. In the field of natural product extraction, process optimization aims to identify the key parameter combinations that maximize both the extractability and the structural fidelity of the targeted phytochemicals [7]. RSM is a statistical optimization technique that establishes a mathematical relationship between response variables and multiple experimental factors through polynomial regression. This method involves constructing a rational experimental design to establish a mathematical model relating process parameters (independent variables) to response indicators (dependent variables), thereby enabling analysis of the effects of individual factors and their interactions on the response. Through response surface analysis, the optimal process region can be identified within a multidimensional parameter space, and model validation ensures the reliability of its predictions. The application of RSM significantly reduces the number of required experiments, lowers research and development costs and time requirements, and achieves systematic process optimization and performance enhancement by focusing on the most critical variables [23,24].

Therefore, leveraging the principles of ultrasound-assisted extraction (where high-frequency pulsed cavitation accelerates mass transfer and promotes rapid flavonoid release) and enzyme-assisted extraction (which uses specific enzymes to disrupt the dense cell-wall network and increase permeability), this study optimized the process parameters for EAU of flavonoids from PN using RSM. To verify the synergistic effect of EAU, the morphological characteristics of the extracted PN residues were compared using SEM. The extracted purified flavonoids were analyzed and characterized using UPLC-Triple-TOF/MS. Furthermore, to elucidate the chemical and physical properties of the purified flavonoids, FTIR and DSC were conducted for functional group identification and thermal stability analysis. Additionally, the in vitro antioxidant and α-GLU- and α-AMY-inhibitory activities of the purified flavonoids were evaluated. Collectively, this study aims to provide a scientific basis for the comprehensive utilization of PN resources.

## 2. Materials and Methods

### 2.1. Materials and Reagents

The test samples of PN were collected in September 2025 from the Liangshui National Nature Reserve (128°53′20″ E, 47°10′50″ N), Dailing District, Yichun City, Heilongjiang Province, China. This region experiences a continental humid monsoon climate, with warm and rainy summers, a mean annual temperature of 0.3 °C, and an average annual precipitation of approximately 676.0 mm. Following collection, the needles were manually cleaned, oven-dried at 45 °C for 24–48 h to constant weight, and then ground and passed through a 60-mesh sieve; the resulting powder was used for subsequent flavonoid extraction.

All chemical reagents used in this study were of analytical grade unless otherwise specified. Absolute ethanol was supplied by Fuyu Fine Chemical Co., Ltd. (Tianjin, China). The following reagents were sourced from Macklin Biochemical Co., Ltd. (Shanghai, China): methanol, acetonitrile, formic acid, dimethyl sulfoxide (DMSO), p-nitrophenyl-α-D-glucopyranoside (PNPG), and acarbose. Kemiou Chemical Reagent Co., Ltd. (Tianjin, China) provided citric acid, aluminum nitrate, hydrochloric acid, and L (+)-ascorbic acid (vitamin C, VC). Sinopharm Chemical Reagent Co., Ltd. (Shanghai, China) supplied sodium nitrite, sodium hydroxide, and 2,6-di-tert-butyl-4-methylphenol (BHT). Yuanye Bio-Technology Co., Ltd. (Shanghai, China) was the source of rutin (analytical standard, HPLC ≥ 98%). Lanji Technology Development Co., Ltd. (Shanghai, China) provided pectinase and cellulase. Additional reagents and their respective suppliers were as follows: AB-8 macroporous adsorption resin from Tianjin Huida Chemical Co., Ltd. (Tianjin, China); α-AMY, 2,2-diphenyl-1-picrylhydrazyl (DPPH) and 2,2′-azino-bis(3-ethylbenzothiazoline-6-sulfonic acid) (ABTS) from BioTope Technology Co., Ltd. (Beijing, China); potassium persulfate from Tianli Chemical Reagent Co., Ltd. (Tianjin, China); sodium dihydrogen phosphate and disodium hydrogen phosphate from Yongda Chemical Reagent Co., Ltd. (Tianjin, China); α-GLU from Yishijiu (Jiangsu Lianyungang) Biotechnology Co., Ltd. (Lianyungang, China); 3,5-Dinitrosalicylic acid (DNS) colorimetric reagent from Wenlai Biotechnology Co., Ltd. (Fuzhou, China); soluble starch from Hengxing Chemical Reagent Manufacturing Co., Ltd. (Tianjin, China); and sodium carbonate and sodium chloride from Guangfu Technology Development Co., Ltd. (Tianjin, China).

### 2.2. Extraction and Determination of Total Flavonoids from PN

**Figure 1:**
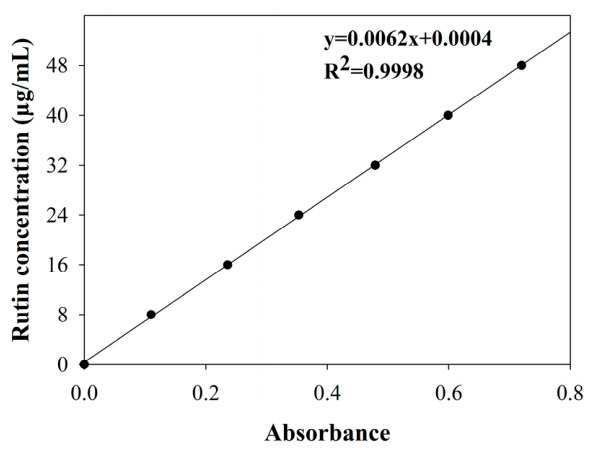
Standard curve for rutin. A linear relationship was found between the concentration of rutin and its absorbance within the range of 0 to 48 μg/mL.

Flavonoid quantification was performed using the NaNO_2_-Al (NO_3_)_3_-NaOH colorimetric assay, with rutin as the external standard [25]. The analytical procedure involved sequential reactions: oxidation with 5% (*w*/*v*) NaNO_2_ for 6 min, complexation with 10% (*w*/*v*) Al (NO_3_)_3_ for another 6 min, and final alkalization with 4% (*w*/*v*) NaOH. The resulting solution was then diluted to 25 mL with 50% (*v*/*v*) ethanol and allowed to stabilize at 22 ± 2 °C for 15 min. Absorbance was measured at 510 nm (T6 New Century UV-Vi’s spectrophotometer, Beijing Puxi General Instrument Co., Ltd., Beijing, China). A six-point rutin calibration curve (8.0–48 μg/mL) was established following the same protocol after appropriate dilution of a 200 μg/mL stock solution (5.00 mg/25 mL in 50% (*v*/*v*) ethanol). Figure 1 presents the calibration curve obtained by plotting absorbance against concentration.

After being accurately weighed, 2.00 g of PN powder was placed in a 250 mL Erlenmeyer flask (Sichuan Shubo (Group) Co., Ltd., Chongzhou, China). A specified volume of citric acid solution (pH 4.5) was added, followed by a defined proportion of cellulase and pectinase. The mixture was thoroughly shaken and subjected to enzymatic pretreatment at a set temperature. Following enzymatic hydrolysis, an appropriate volume of anhydrous ethanol was introduced, and the resulting mixture was subjected to ultrasound irradiation in a JP-180st bath-type sonicator (Shenzhen Jiemeng Technology Co., Ltd., Shenzhen, China) under controlled power and time conditions. The instrument was operated at a fixed frequency of 40 kHz with a tank capacity of 53 L and a heating range of 0–80 °C. Subsequently, the extracts of PN from each group were transferred into centrifuge tubes and centrifuged at 4000 rpm for 10 min using a TD5A-WS high-speed centrifuge (Hunan Xiangyi Laboratory Instrument Development Co., Ltd., Changsha, China). The collected supernatants were used for *TFC* analysis according to the colorimetric procedure described above. The *TFC* was quantified as rutin equivalents and expressed in milligrams per gram of dry matter (mg rutin/g dry matter), based on the equation *TFC* (mg rutin/g dry matter) = (*C* × *N* × *V*)/*M*, where *C* denotes the flavonoid concentration (mg/mL) derived from the rutin calibration curve (Figure 1), *N* is the dilution factor applied, *V* refers to the total extract volume (mL), and *M* corresponds to the dry weight of the sample (g). All measurements were performed in triplicate, and the results were expressed as the mean ± standard deviation (SD).

### 2.3. Single-Factor Experiment Design

Single-factor experiments were conducted to evaluate the effects of key parameters influencing enzymatic hydrolysis and ultrasonic efficiency. The investigated factors and their tested levels were as follows: cellulase-to-pectinase ratio (3:1, 2:1, 1:1, 1:2, 1:3), enzyme dosage (0.5, 1, 1.5, 2, 2.5, wt%, based on the dry weight of PN), enzymatic hydrolysis time (1 h, 1.5 h, 2 h, 2.5 h, 3 h), enzymatic hydrolysis temperature (42.5 °C, 45 °C, 47.5 °C, 50 °C, 52.5 °C), ultrasonication time (10 min, 15 min, 20 min, 25 min, 30 min), ultrasonic power (0 W, 150 W, 300 W, 450 W, 600 W), liquid-to-solid ratio (20:1, 30:1, 40:1, 50:1, 60:1, mL/g, based on the dry weight of PN), and ethanol concentration (40%, 50%, 60%, 70%, 80%, *v*/*v*). When examining the multilevel effect of one factor, all other factors were held constant at the following baseline conditions: cellulase-to-pectinase ratio of 1:1, enzyme dosage of 1% (w/PN w), enzymatic hydrolysis time of 2 h, enzymatic hydrolysis temperature of 45 °C, liquid-to-solid ratio of 40:1 mL/g, ethanol concentration of 60% (*v*/*v*), ultrasonication time of 20 min, and ultrasonic power of 300 W. Flavonoid compounds were extracted according to the above design and subsequently determined. All measurements were performed in triplicate, and the results are presented as mean values.

### 2.4. RSM Experimental Design

Guided by the outcomes of the preliminary single-factor screening, three independent variables exerting the most pronounced influence on *TFC* were advanced to response surface optimization, each examined at three coded levels (−1, 0, +1). A 17-run Box–Behnken design (BBD) was constructed with the aid of Design-Expert 13.0 (Stat-Ease, Inc., Minneapolis, MN, USA), incorporating twelve factorial combinations and five replicate center points to provide an estimate of pure experimental error. Upon completion of the designed trials, the experimental data were fitted to a second-order polynomial model through multiple regression. The general form of the fitted quadratic equation relating the response (Y) to the independent variables is as follows:

Y = β_0_ + Σ β_i_ X_i_ + Σ β_ii_ X_i_^2^ + Σ β_ij_ X_i_ X_j_

where Y denotes the predicted response; β_0_, β_i_, β_ii_, and β_ij_ represent the constant, linear, quadratic, and interaction regression coefficients, respectively; and X_i_ and X_j_ are the coded independent variables. Analysis of variance (ANOVA) was subsequently applied to evaluate the statistical significance of the model terms and to assess the adequacy of the fitted surface.

Confirmatory trials were performed using the optimal conditions predicted by the Design-Expert 13.0 software. To delineate the individual contributions of enzymatic hydrolysis and ultrasonic treatment to the total flavonoid yield from PN, three control groups were established under otherwise identical processing conditions: CE (without enzyme and ultrasound), enzyme-assisted ethanol extraction without ultrasonic treatment (EE) and ultrasound-assisted ethanol extraction without enzyme pretreatment (UE). The extraction yields of the EAU optimized group were compared with those of the three control groups to assess the individual and combined contributions of the enzymatic and ultrasonic treatments. Three replicate verification experiments were conducted using the optimal parameters derived from the response surface analysis, yielding the highest *TFC* from PN.

### 2.5. SEM of PN Residues

To further investigate the effects of enzymatic hydrolysis combined with ultrasonic extraction on the microstructure of PN residues, the samples obtained after flavonoid extraction were sputter-coated with gold and examined using a Zeiss Sigma 300 scanning electron microscope (Carl Zeiss, Oberkochen, Germany) equipped with an energy-dispersive spectrometer (Oxford Xplore 30, Oxford Instruments, Abingdon, Oxfordshire, UK).

### 2.6. Purification of Flavonoids from PN

Crude flavonoid extracts obtained under the optimized conditions were subjected to purification by AB-8 macroporous resin column chromatography. The resin was activated prior to use according to a reported protocol [25], with the following adjustments: Briefly, the adsorbent was fully swollen in 95% (*v*/*v*) ethanol over a 24 h period and then washed thoroughly with distilled water to eliminate residual solvent. Alkaline and acid treatments were sequentially performed by immersing the resin in four bed volumes of 5% (*w*/*v*) NaOH and 5% (*v*/*v*) HCl, respectively, each for 5 h with intermediate and final water rinses until the effluent reached neutral pH. After gravitational settling for 1 h, the prepared resin was slurry-packed into a glass column (height-to-diameter ratio = 10:1) to produce a packed bed of approximately 2.2 L. The sample was loaded onto the column at a volume corresponding to one-fourth of the bed volume and a flow rate of 1.0 mL/min. Following a static adsorption period of 4 h, the column was washed with two bed volumes of distilled water to remove unbound or weakly retained impurities. The adsorbed flavonoids were then eluted with 70% (*v*/*v*) ethanol at 2.0 mL/min. The eluate was concentrated to near dryness on an EYELA N-1200B rotary evaporator (Shanghai Ailang Instrument Co., Ltd., Shanghai, China) and subsequently freeze-dried using an EYELA FDU-2110 lyophilizer (Tokyo Rikakikai Co., Ltd., Tokyo, Japan). The purified flavonoid powder was weighed and stored for further characterization.

### 2.7. UPLC-Triple-TOF/MS Composition Analysis

The lyophilized material was pulverized to a fine powder using a bead mill operated for six grinding cycles. A 50 mg portion of the resultant powder was weighed and extracted with 500 μL of ethanol solution under ultrasonication for 30 min. The suspension was clarified by centrifugation (17,000× *g*, 10 min, 20 °C), and the clear supernatant was collected into an autosampler vial for subsequent instrumental analysis.

The analysis was performed using a UHPLC-HRMS system, which consisted of an H-Class UHPLC system (Waters Corporation, Milford, MA, USA) coupled with a 6600 Triple-TOF high-resolution mass spectrometer (AB SCIEX, Framingham, MA, USA). Separation was achieved on an ACQUITY UPLC HSS T3 column (1.8 μm, 2.1 × 100 mm; Waters Corporation, Milford, MA, USA). The mobile phase comprised two components: 0.1% formic acid in water (A) and 0.1% formic acid in acetonitrile (B). The column temperature was maintained at 40 °C, and the injection volume was 4 μL. The detailed gradient elution program is provided in Table 1.

High-resolution mass spectrometric analysis was carried out on an AB 6600 Triple-TOF system (AB Sciex, Framingham, MA, USA) operated in Information-Dependent Acquisition (IDA) mode. Instrument control and data collection were managed through Analyst TF 1.7 software. In each duty cycle, precursor ions exhibiting signal intensities above a threshold of 100 counts were automatically subjected to MS/MS fragmentation. The MS^1^ scan range was set at *m*/*z* 50–1200. The collision energy was set to 30 eV, and up to 15 product ion spectra were collected per cycle. The electrospray ionization (ESI) source parameters were configured as follows: nebulizing gas pressure (GS1), 60 psi; auxiliary gas pressure, 60 psi; curtain gas pressure, 35 psi; source temperature, 550 °C; and ion spray voltage, 5500 V for positive ion mode or −4500 V for negative ion mode.

### 2.8. FTIR and DSC Analysis

Infrared spectra of the purified flavonoid extract (Section 2.6) were recorded on a Nicolet iS50 FT-IR spectrometer (Thermo Fisher Scientific, Waltham, MA, USA). The lyophilized sample was homogenized with potassium bromide (KBr) and compressed into a transparent disc. Spectra were acquired in the 4000–400 cm^−1^ region at a spectral resolution of 4 cm^−1^, with a data spacing of 4.4 cm^−1^ and an optical path velocity of 0.4747 cm/s.

DSC was conducted on an HSC-1 instrument (Beijing Hengjiu Experimental Equipment Co., Ltd., Beijing, China) to assess the thermal properties of the lyophilized flavonoid extract (Section 2.6). A weighed portion of the sample was hermetically sealed in an aluminum pan and heated from ambient temperature to 250 °C at a heating rate of 10 °C/min.

### 2.9. In Vitro Bioactivity Assays

#### 2.9.1. DPPH Radical Scavenging Activity Assay

The DPPH radical scavenging assay was performed with slight modifications based on the method described by Chen et al. [26]. At room temperature, 100 µL of the appropriately diluted sample was pipetted into individual wells of a 96-well microplate, and an equal volume of 0.30 mmol/L DPPH working solution (freshly prepared in methanol) was added. The plate was agitated on a plate shaker for 30 s to ensure complete mixing, and then it was kept in darkness at ambient temperature for 30 min. The absorbance (A_s_) of each well was recorded at 517 nm using an AMR-100T microplate reader (Hangzhou Ausheng Instrument Co., Ltd., Hangzhou, China). The freeze-dried flavonoid sample was serially diluted using 70% (*v*/*v*) methanol via a two-fold dilution method to obtain sample solutions at concentrations of 10, 25, 50, 100, 200, 400, 800, and 1600 µg/mL. VC and BHT were separately diluted with 70% (*v*/*v*) methanol to prepare positive control solutions at concentrations of 2, 4, 8, 16, 32, 64, and 128 µg/mL. The blank control (A_0_) consisted of 70% (*v*/*v*) methanol in place of the sample, while the sample background control (A_b_) contained the sample solution and 70% (*v*/*v*) methanol without DPPH. All determinations were carried out in triplicate. The percentage of DPPH radical inhibition was calculated using the following equation:

DPPH scavenging (%) = [1 − (A_s_ − A_b_)/A_0_] × 100%

where A_s_, A_b_, and A_0_ denote the absorbance of the sample, sample background, and blank control, respectively. The half-maximal inhibitory concentration (IC_50_) was determined by nonlinear regression analysis using GraphPad Prism version 10.6 and expressed in µg/mL.

#### 2.9.2. ABTS Cation Radical Scavenging Activity Assay

The ABTS cation radical scavenging assay was performed with slight modifications based on the method described by Chen et al. [26]. A stock ABTS solution was prepared by dissolving 38.4 mg of ABTS and 6.6 mg of potassium persulfate in distilled water and adjusting the final volume to 10 mL. The mixture was allowed to react in the dark at room temperature for 12–16 h. The stock solution was then diluted with 0.1 M PBS (pH 7.4) until the absorbance at 734 nm reached 0.70 ± 0.02 to obtain the ABTS working solution. At room temperature, 20 µL of the sample solution was added to a 96-well microplate, after which 180 µL of the ABTS working reagent was added. The plate was shaken briefly for mixing and then held in the dark at ambient temperature for 6 min, after which the absorbance (A_s_) was recorded at 734 nm. The concentration gradients for the flavonoid sample solutions and positive control solutions were prepared exactly as outlined for the DPPH assay. The blank control (A_0_) was set up with 70% (*v*/*v*) methanol in place of the sample. The sample background control (A_b_) was prepared by replacing the ABTS working solution with 70% (*v*/*v*) methanol. All experiments were performed in triplicate. The scavenging percentage and IC_50_ were computed using the same equation and regression method described in the DPPH section.

#### 2.9.3. α-Glucosidase-Inhibitory Activity Assay

The α-GLU-inhibitory activity assay was performed with modifications based on the method described by Zhao et al. [27]. At room temperature, 50 µL of the sample solution was added to a 2 mL centrifuge tube, followed by 50 µL of α-GLU solution (5 U/mL). After incubation at 37 °C for 10 min, 50 µL of 5 mM PNPG solution was added and thoroughly mixed. After an additional 30 min of incubation, the enzymatic reaction was quenched by the addition of 750 µL of Na_2_CO_3_ solution. Immediately, 100 µL of the reaction mixture was transferred to a 96-well microplate, and the absorbance (A_s_) was measured at 405 nm. The freeze-dried flavonoid powder was dissolved in DMSO to prepare a 25 mg/mL stock solution, which was subsequently diluted with 0.1 M phosphate-buffered saline (PBS) (pH 6.8) to obtain concentration gradients of 10, 25, 50, 100, 200, 400, 800, and 1600 µg/mL. The positive control, acarbose, was diluted with the same PBS buffer to final concentrations of 0.5, 1, 2, 4, 8, and 16 µM. Both the α-GLU and PNPG solutions were prepared using PBS (0.1 M, pH 6.8). The negative control (A_0_) was obtained by replacing the sample with PBS (0.1 M, pH 6.8), the sample background control (A_b_) by replacing the α-GLU with PBS (0.1 M, pH 6.8), and the reagent blank control (A_r_) by replacing both the sample and α-GLU with PBS (0.1 M, pH 6.8). All tests were performed in triplicate. The α-GLU inhibition rate (%) was calculated as [1 − (A_s_ − A_b_)/(A_0_ − A_r_)] × 100%, and the IC_50_ value was determined accordingly.

#### 2.9.4. α-Amylase-Inhibitory Activity Assay

The α-AMY-inhibitory activity assay was performed with modifications based on the method described by Zhao et al. [27]. A total of 40 µL of the sample solution was added to a 96-well microplate, followed by 20 µL of α-AMY solution (1 U/mL). After incubation at 37 °C for 10 min, 40 µL of 1% (*w*/*v*) pre-gelatinized starch solution was added and thoroughly mixed, and the reaction was continued at 37 °C for another 10 min. Subsequently, 100 µL of DNS reagent was added to terminate the reaction. The microplate was then placed on a tray and boiled in a water bath for 10 min. After cooling to room temperature, the absorbance (A_s_) was measured at 540 nm. The freeze-dried flavonoid powder was dissolved in 70% (*v*/*v*) methanol to prepare a 10 mg/mL stock solution, which was then diluted with 0.02 M PBS (pH 6.9) to obtain concentration gradients of 0.5, 1, 2, 4, and 8 mg/mL. The positive control, acarbose, was diluted with the same PBS buffer to final concentrations of 0.5, 1, 2, 4, and 8 µM. Both the α-AMY and gelatinized starch solutions were prepared using the PBS (0.02 M, pH 6.9). The negative control (A_0_) was obtained by replacing the sample with PBS (0.02 M, pH 6.9), the sample background control (A_b_) by replacing the α-AMY with PBS (0.02 M, pH 6.9), and the reagent blank control (A_r_) by replacing both the sample and α-AMY with PBS (0.02 M, pH 6.9). All tests were performed in triplicate. The α-AMY inhibition rate and IC_50_ value were calculated using the same formula as described for the α-GLU-inhibitory activity assay.

### 2.10. Statistical Analysis

All experiments were carried out in triplicate, and results were expressed as the mean ± standard deviation (SD). Group differences were examined by one-way analysis of variance (ANOVA) followed by Duncan’s post hoc test, with significance set at *p* < 0.05. Statistical computations were executed using IBM SPSS Statistics 26.0. Response surface design and regression modeling were performed using Design-Expert 13.0 (Stat-Ease, Inc., Minneapolis, MN, USA), and graphical representations were generated in Sigma Plot 12.5.

## 3. Results and Discussion

### 3.1. Results and Analysis of Single-Factor Experiments

A single-factor experiment is an experimental design in which only one factor is investigated at a time, in order to evaluate its effect on the experimental results. As the most fundamental approach in experimental design, this methodology aims to explore the variation pattern between a single controllable factor and the response variable across different treatment levels. Moreover, it provides a basis for subsequent multi-factor interaction optimization [28]. The plant cell wall is primarily composed of lignin, cellulose, pectin, and hemicellulose. Enzymes are highly specific and can efficiently disrupt this complex cell-wall structure under mild conditions, thereby improving the efficiency of conventional extraction methods. Cellulase hydrolyzes β-1,4 glycosidic bonds, disrupts the crystalline structure of cellulose, and weakens the overall rigidity of the cell wall [29]. Pectinase degrades pectin and dissolves the middle lamella, leading to tissue dissociation and cell separation, which increase the solvent-accessible surface area [30,31]. Consequently, key parameters influencing the efficacy of enzymatic pretreatment and ultrasonic extraction were selected for investigation, including the cellulase-to-pectinase ratio, enzyme dosage, enzymatic hydrolysis time, enzymatic hydrolysis temperature, ultrasonication time, ultrasonic power, liquid-to-solid ratio, and ethanol concentration.

#### 3.1.1. Effect of Enzyme Ratio on the *TFC* from PN

**Figure 2:**
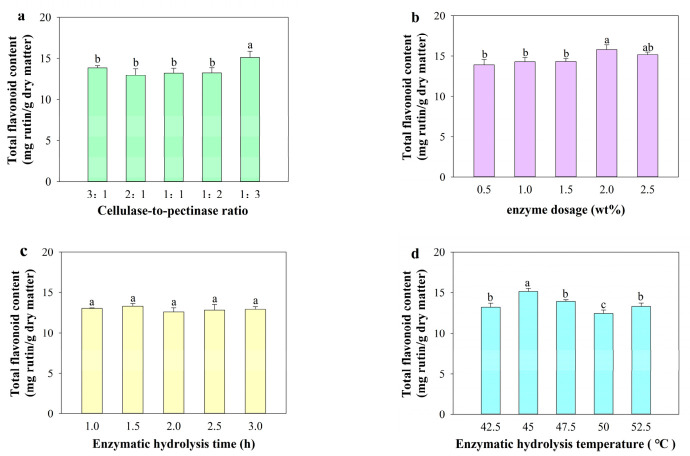

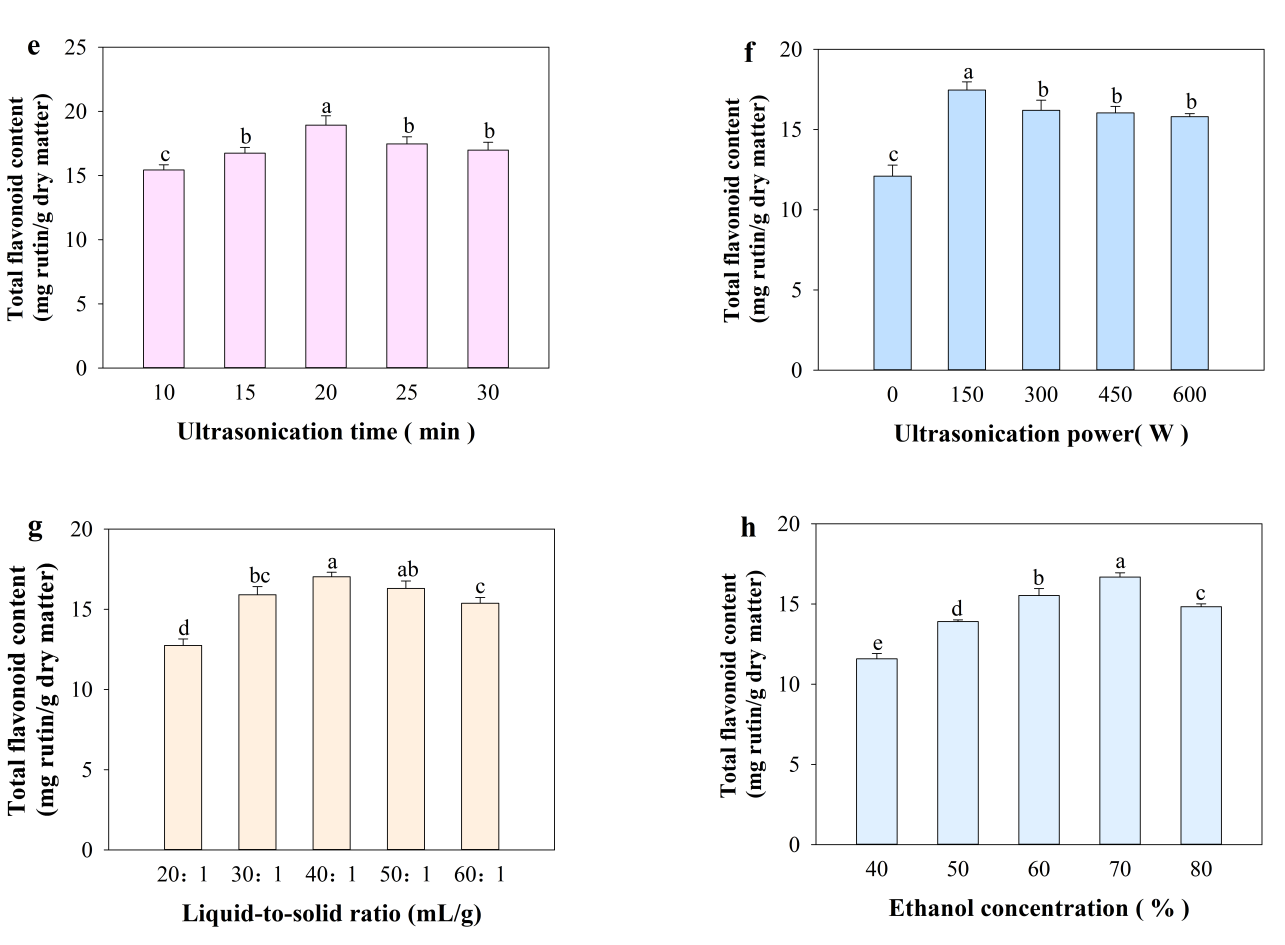
Single-factor experimental analysis of *TFC*. Effects of different extraction conditions on *TFC*: (**a**) cellulase-to-pectinase ratio, (**b**) enzyme dosage, (**c**) enzymatic hydrolysis time, (**d**) enzymatic hydrolysis temperature, (**e**) ultrasonication time, (**f**) ultrasonic power, (**g**) liquid-to-solid ratio, and (**h**) ethanol concentration. Data are presented as means ± standard deviations (*n* = 3). Different letters indicate significant differences.

Figure 2a depicts the influence of the enzyme ratio on yield under a constant 1.0% total enzyme addition. The optimal performance emerged at a cellulase-to-pectinase ratio of 1:3, where the *TFC* reached 15.12 ± 0.80 mg rutin/g dry matter. Relative to other tested ratios, this specific composition conferred a significant enhancement in extraction efficiency. This result can be attributed to the high pectin content in the middle lamella and primary cell walls of PN, which constitutes the primary and most robust barrier to solvent penetration [32]. Consequently, a higher proportion of pectinase is required for more extensive enzymatic hydrolysis of the cell wall, thereby enhancing solvent permeability and facilitating the release of flavonoid compounds. Based on this finding, a cellulase-to-pectinase ratio of 1:3 was selected for the subsequent RSM optimization experiments.

#### 3.1.2. Effect of Enzyme Dosage on the *TFC* from PN

The synergistic application of cellulase and pectinase compromises cell-wall integrity and augments membrane porosity, resulting in a marked improvement in flavonoid extraction efficiency [31]. The effect of mixed-enzyme dosage on the *TFC* is shown in Figure 2b. The *TFC* increased gradually with increasing enzyme dosage. The maximum yield (15.84 ± 0.59 mg rutin/g dry matter) was reached at an enzyme dosage of 2.0 wt%. Beyond this point, further increases in enzyme dosage did not lead to significant improvements in flavonoid extraction. This phenomenon is likely due to the low enzyme levels and the insufficient contact between the enzymes and the PN cell walls, resulting in limited hydrolysis and incomplete cell-wall disruption, which consequently yields lower flavonoid extraction [33]. When the enzyme dosage reached 2.0%, the enzymes sufficiently contacted and hydrolyzed the cell-wall components, leading to more complete structural breakdown. Further increasing the enzyme amount beyond this saturation point did not enhance the release of flavonoids. Therefore, a mixed-enzyme dosage of 2.0% was selected for subsequent RSM optimization experiments.

#### 3.1.3. Effect of Enzymatic Hydrolysis Time on the *TFC* from PN

Enzymatic hydrolysis time is a critical factor that determines the extent to which enzymes can exert their catalytic potential. Figure 2c illustrates the influence of enzymatic hydrolysis duration on the *TFC* extracted from PN. The yield increased progressively with time and attained a maximum at 2 h; however, the *TFC* did not differ significantly when the hydrolysis time exceeded 1 h. This indicates that a 1 h enzymatic treatment was sufficient to achieve near-complete hydrolysis of the pine needle cell walls. Shorter hydrolysis durations resulted in insufficient enzymatic action, leading to incomplete cell-wall disruption and, consequently, lower flavonoid extraction [34]. Therefore, considering both extraction efficiency and time cost, an enzymatic hydrolysis time of 1.0 h was selected for subsequent RSM experiments.

#### 3.1.4. Effect of Enzymatic Hydrolysis Temperature on the *TFC* from PN

Enzymatic hydrolysis temperature is a key parameter that directly influences enzyme reaction kinetics, stability, and ultimately, the efficiency of cell-wall degradation. As shown in Figure 2d, the *TFC* from PN increased with temperature and reached a maximum of 15.14 ± 0.43 mg rutin/g dry matter at 45 °C; beyond this temperature, the yield exhibited a noticeable decline. This trend can be attributed to the balance between enzymatic activity and thermal denaturation. Elevated temperatures may suppress enzyme activity; in addition, they may facilitate the degradation (e.g., oxidation) or isomerization of flavonoid compounds, resulting in a decreased apparent flavonoid content [1,28]. Conversely, at temperatures below 45 °C, the kinetic energy of the enzyme–substrate system is suboptimal, resulting in reduced catalytic activity and slower hydrolysis rates. Consequently, the disruption of the lignocellulosic structure of PN is less complete, which limits the release of intracellular flavonoids [35]. The temperature of 45 °C likely represents the optimal compromise where the activities of both cellulase and pectinase approach their maxima without significant loss of enzyme stability over the hydrolysis period. Therefore, considering both the maximization of enzymatic efficiency and the preservation of enzyme stability, an enzymatic hydrolysis temperature of 45 °C was selected for subsequent RSM optimization experiments.

#### 3.1.5. Effect of Ultrasonication Time on the *TFC* from PN

The extraction of bioactive constituents from plant materials typically requires sufficient contact time for the mass transfer process to approach dynamic equilibrium. As illustrated in Figure 2e, the *TFC* rose progressively with ultrasonication time up to 20 min, where a maximum of 18.93 ± 0.73 mg rutin/g dry matter was attained, after which a gradual decline was observed. Further extension of the ultrasonication time led to a gradual decline in the extraction yield. The observed trend arises from the competing effects of ultrasonication duration. Shorter times (<20 min) provide insufficient cavitation energy to fully disrupt cells and achieve mass transfer equilibrium, limiting extraction. At 20 min, cavitation forces optimally degrade cellular barriers, maximizing flavonoid release. Beyond this point, prolonged exposure to intense cavitation promotes the degradation of heat-sensitive flavonoids and increases impurity co-extraction, thereby lowering the net yield [36]. Therefore, considering both extraction efficiency and compound stability, an ultrasonication time of 20 min was selected for subsequent RSM optimization experiments.

#### 3.1.6. Effect of Ultrasonic Power on the *TFC* from PN

The effects of different ultrasonic power levels on the extraction yield of total flavonoids from PN are shown in Figure 2f. The highest *TFC* (17.46 ± 0.51 mg rutin/g dry matter) was achieved at an ultrasonic power of 150 W. A gradual improvement in *TFC* was observed as the ultrasonic power was raised from 0 to 150 W. This trend is largely accounted for by the intensified acoustic cavitation generated within the extraction medium at higher power levels. The local high temperature, high pressure, and intense shear forces produced during the collapse of the cavitation bubbles effectively disrupted the cell wall and cell membrane structures of the PN, thereby increasing solvent permeability and accelerating the mass transfer rate of flavonoids from the intracellular space to the solvent, which ultimately improved the extraction efficiency [37,38]. However, when the ultrasonic power exceeded 150 W, the extraction yield of total flavonoids gradually decreased. This decline was likely due to excessive and overly intense cavitation effects at higher ultrasonic powers. The resulting extreme physical conditions may induce oxidation or structural degradation of heat-sensitive flavonoid compounds. Moreover, the vigorous mechanical action can excessively disrupt the plant tissue, leading to the leaching of large amounts of non-target impurities such as polysaccharides, proteins, and pigments, which interfere with the determination of flavonoids in the extract and, consequently, reduce the measured *TFC* [39]. Therefore, the ultrasonic power was fixed at 150 W in the RSM experimental design that followed.

#### 3.1.7. Effect of Liquid-to-Solid Ratio on the *TFC* from PN

The *TFC* is governed by the partitioning equilibrium of solutes between the plant matrix and the extraction medium. To examine this relationship, five liquid-to-solid ratios (20:1, 30:1, 40:1, 50:1, and 60:1) were evaluated. As presented in Figure 2j, the *TFC* rose steadily as the solvent proportion increased, attaining a maximum of 17.03 ± 0.28 mg rutin/g dry matter at 40:1. At lower ratios (<40:1), the available solvent volume was merely adequate to moisten the surface of the needle powder, limiting further mass transfer. Under such conditions, the flavonoid components inside the material required a longer and more tortuous solid-phase diffusion path to reach the surface. Moreover, local saturation of the solvent occurred, which halted further diffusion, resulting in a substantial amount of flavonoids remaining in the residue [7,40]. When the liquid-to-solid ratio exceeded 40:1, the *TFC* showed a decreasing trend. This decline can be attributed to the excessive solvent volume during ultrasound-assisted extraction, which led to the leaching of large amounts of non-target impurities and altered the rheological properties of the extraction system, thereby weakening the intensity of ultrasonic cavitation. Consequently, not only did the yield of total flavonoids decrease, but solvent efficiency was also compromised. Moreover, excessive solvent volumes led to the co-extraction of interfering matrix constituents, adding to the complexity and expense of downstream purification steps [41]. Hence, a solvent-to-feed ratio of 40:1 (mL/g) was chosen as the fixed parameter for the ensuing RSM trials.

#### 3.1.8. Effect of Ethanol Concentration on the *TFC* from PN

Water, as a solvent, promotes the swelling of plant cells, softens the cell-wall structure, and increases intracellular osmotic pressure, thereby driving the outward diffusion of intracellular components. In contrast, ethanol disrupts the phospholipid bilayer of the cell membrane, enhances its permeability, and induces partial denaturation of membrane proteins, thereby more thoroughly breaking down the mass transfer barriers between the intracellular and extracellular environments [42]. The influence of ethanol concentration on the *TFC* was examined, with the corresponding data displayed in Figure 2h. As the ethanol concentration increased from 40% to 70%, the *TFC* gradually increased, reaching its highest value (16.68 ± 0.26 mg rutin/g dry matter) at 70% ethanol. Previous studies have shown that the polarity range of 50–70% (*v*/*v*) aqueous ethanol covers the optimal solubility range of most common flavonoids, from relatively polar glycosides to moderately polar aglycones, thereby maximizing the *TFC* within this concentration range [42]. At an ethanol concentration of 70%, the solvent polarity achieved the best match with the polarity of the majority of the flavonoid components, resulting in the highest extraction yield [43]. However, when the ethanol concentration was further increased to 80%, the solvent polarity decreased, reducing its dissolving capacity for highly polar flavonoid glycosides and, consequently, leading to a decrease in the *TFC* [44]. Therefore, an ethanol concentration of 70% was selected for subsequent optimization experiments using RSM.

### 3.2. Optimization of Total Flavonoid Extraction from PN Using RSM

#### 3.2.1. Model Fitting and Statistical Analysis

Single-factor experiments only reveal the effects of individual variables on the extraction content, yet interactions among different factors also significantly influence it. Owing to synergistic effects among variables, RSM generally outperforms conventional single-factor optimization in the field of natural compound extraction. Three factors (enzyme dosage, ultrasonication time, and ethanol concentration) were selected for further optimization according to the single-factor test results and the BBD principle. Table 2 lists the factor levels for the RSM experimental design.

A three-factor, three-level BBD was constructed using Design-Expert 13.0 (Stat-Ease, Inc., Minneapolis, MN, USA). The experimental matrix consisted of 17 runs, including five center-point replicates to provide an estimate of pure experimental error; the experimental results are summarized in Table 3. The data in Table 3 were subjected to quadratic polynomial fitting using Design-Expert 13.0, followed by ANOVA of the model. The fitted quadratic regression equation can be expressed as follows:

TFC = 16.37 − 0.5405A + 0.4844B + 0.1471C − 0.0803AB − 0.0809AC + 0.3385BC − 0.4456A^2^ −1.92 B^2^ − 1.13C^2^

The ANOVA for the fitted quadratic model is summarized in Table 4. The model yielded an *F*-value of 37.04 (*p* < 0.0001), confirming that the regression was highly significant and suitable for describing the extraction of flavonoids from PN [45]. The non-significant lack of fit (*p* = 0.6960 > 0.05) further validated the adequacy of the model in representing the experimental data. The precision and reliability of the model were assessed through the coefficient of variation (*C.V.* = 1.95%) and the coefficient of determination (*R*^2^ = 0.9794). The adjusted *R*^2^ of 0.9530 indicated that the selected independent variables accounted for 95.30% of the total variance in the *TFC*, reflecting a strong agreement between the predicted and observed values, with minimal experimental error. Comparison of the *F*-values revealed that the relative impact of the three factors on the *TFC* was as follows, in descending order: enzyme dosage (A) > ultrasonication time (C) > ethanol concentration (B). Regarding the significance of individual model terms, the linear coefficient of factor A was highly significant (*p* < 0.001), as were the quadratic terms *B*^2^ and *C*^2^. The linear term B and the quadratic term *A*^2^ also reached statistical significance at the *p* < 0.05 level; however, the interaction terms *AB*, *AC*, and *BC* did not show any significant effects (*p* > 0.05). Based on the above analysis, the established model possesses high accuracy and reliability, and it can be used for the analysis and prediction of the *TFC* from PN.

#### 3.2.2. Analysis of Response Surface

**Figure 3:**
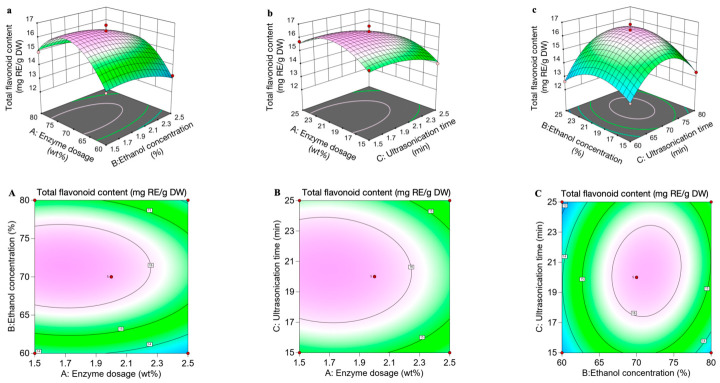
3D (**a**–**c**) and contour (**A**–**C**) response surface plots of interaction effects between enzyme dosage, ethanol concentration, and ultrasonication time on the *TFC* of PN.

Figure 3 illustrates the effects of enzyme dosage, ultrasonication time, and ethanol concentration on the TFC from PN using 3D response surface and contour plots. All three-dimensional response surfaces exhibited a downward-opening characteristic, indicating the existence of a maximum value for the TFC extracted from PN. The response surface plots not only visually demonstrate each factor’s effect on the response value but also reveal the patterns of interaction between factors. In the 3D response surface plots, a steeper slope corresponds to a stronger effect of the factor on the response value. The shape of the contour plots indicates the strength of the interaction between two factors, where more elliptical contours imply a stronger interaction. Conversely, a flat surface with sparse, nearly circular contours indicates weak variable influence and a lack of significant interaction [46]. Specifically, the surface slopes in Figure 3A,a, as well as Figure 3B,b, are relatively flat, with sparse contour lines, indicating that the interactive effects between enzyme dosage and ethanol concentration, and between enzyme dosage and ultrasonication time, on the response value were not significant. While Figure 3C exhibits a steeper surface slope, the nearly circular contours in Figure 3c suggest a lack of significant interactive effect between ethanol concentration and ultrasonication time. These analytical findings align well with the statistical data in Table 4.

#### 3.2.3. Model Validation

Numerical optimization performed with Design-Expert 13.0 yielded the following theoretical optimum: enzyme dosage 1.685% (*w*/*w*, based on PNs’ dry weight), ethanol concentration 71.483% (*v*/*v*), and ultrasonication time 20.547 min; the corresponding predicted *TFC* was 16.585 mg/g. For practical feasibility, these parameters were rounded to an enzyme dosage of 1.7%, an ethanol concentration of 70% (*v*/*v*), and an ultrasonication time of 21 min, while other factors were maintained at the levels pre-selected from the single-factor experiments (enzyme ratio, 1:3; liquid-to-solid ratio, 40:1; enzymatic hydrolysis temperature, 42.5 °C; hydrolysis duration, 1 h; ultrasonication temperature, 50 °C; ultrasonic power, 150 W). Triplicate verification runs conducted under the adjusted conditions afforded a mean flavonoid recovery of 17.0795 ± 0.37 mg/g, which was in close agreement with the model prediction, thereby corroborating the reliability and predictive capability of the fitted model.

**Figure 4:**
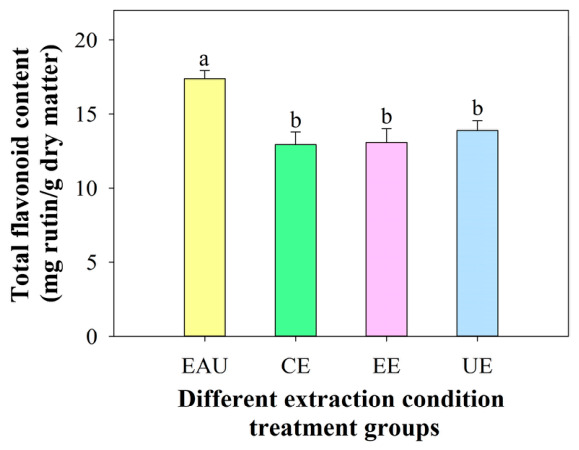
The *TFC* extracted from PN by four extraction methods. The different small letters show significant differences between groups (*p* < 0.05).

In contrast, the *TFC* obtained from PN in the CE group was only 12.9665 mg/g. The EE group yielded 13.1099 mg/g, while the UE group yielded 13.9043 mg/g. As clearly shown in Figure 4, the optimized EAU significantly enhanced the recovery of flavonoids from PN. Similarly, in a study on the extraction of total flavonoids from *Phyllanthus emblica* L., Xiong et al. [47] employed ultrasound-assisted enzymatic hydrolysis (UEAE) combined with Plackett–Burman and response surface methodology (RSM) to optimize the extraction process. Under optimal conditions, the total flavonoid yield reached 10.04 ± 0.40%, which was 65% higher than that obtained by the conventional thermal reflux method. Zhang et al. [48] used ultrasound-assisted enzymatic hydrolysis to extract two bioactive flavonoids (luteolin and apigenin) from celery. After process optimization, the extraction yields reached 42.5 mg/g and 25.3 mg/g, respectively, representing a 26.1-fold and 32.2-fold increase compared to the control group (water extraction without enzyme or ultrasound assistance). Chu et al. [49] optimized the enzyme-assisted ultrasound extraction of flavonoids from *Abelmoschus manihot* (L.). Under the optimal conditions (cellulase-to-pectinase mass ratio of 1:1, enzyme concentration of 3%, pH 6.0, a solvent mixture of 70% ethanol and 0.1 mol/L NaH_2_PO_4_ buffer, ultrasonic power of 500 W, extraction time of 40 min, and temperature of 50 °C), the flavonoid yield reached 3.46 ± 0.012% (*w*/*w*), which was 1.13-fold higher than that obtained by ultrasound-assisted extraction alone (3.06 ± 0.006%). Collectively, these findings consistently demonstrate that the synergistic effect of enzyme and ultrasound significantly improves the extraction efficiency of flavonoids, representing an efficient and environmentally friendly extraction strategy.

### 3.3. SEM Analysis

**Figure 5:**
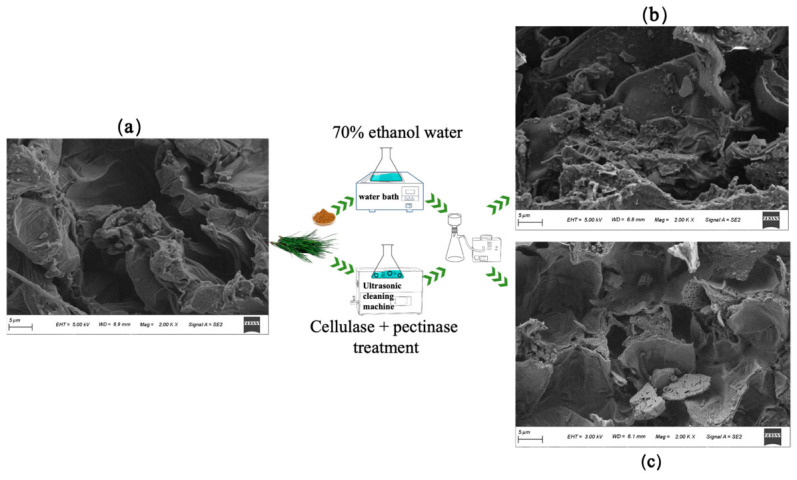
SEM images of PN tissues: (**a**) Lyophilized powder of untreated PN. (**b**) Lyophilized residue of CE. (**c**) Lyophilized residue of EAU.

As shown in Figure 5a, the outer surface of the PN powder was relatively smooth and intact, with the original structure well preserved. The surface of the lyophilized residue from the CE group showed slight wrinkling and particulate matter (Figure 5b). In contrast, the needle surface in the EAU group (Figure 5c) was markedly roughened, fragmented, and eroded. The overall structure became loose, suggesting disruption of the internal fibrous tissue. Numerous stomata-like cavities were observed across the surface, which may have resulted from the rupture of oil-containing tissues. These observations are in line with the earlier literature [50,51].

### 3.4. UPLC-Triple-TOF/MS Analysis and Identification of Flavonoids from PN

#### 3.4.1. Qualitative Analysis of Flavonoid Compounds in PN

**Figure 6:**
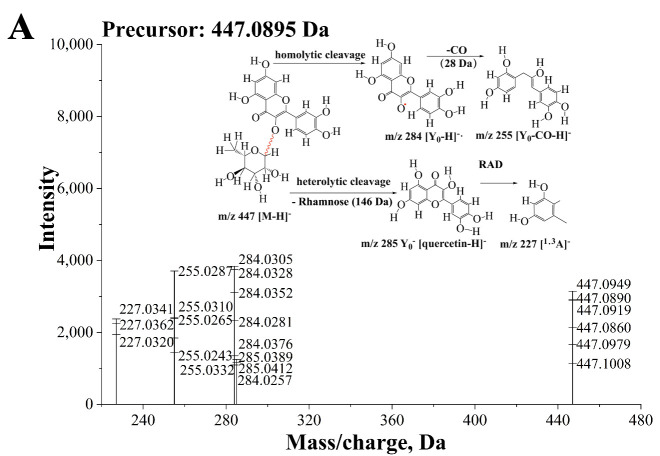

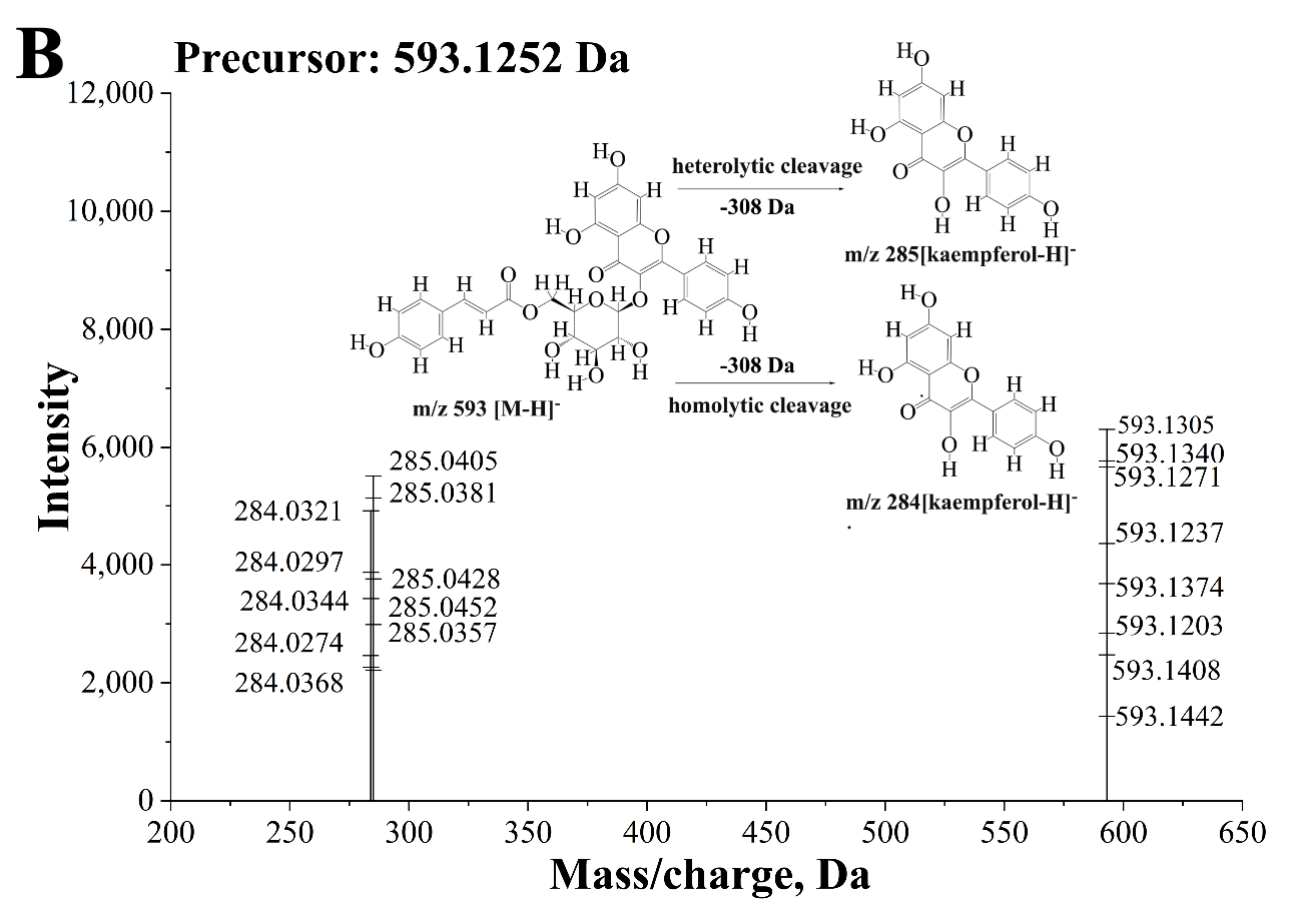

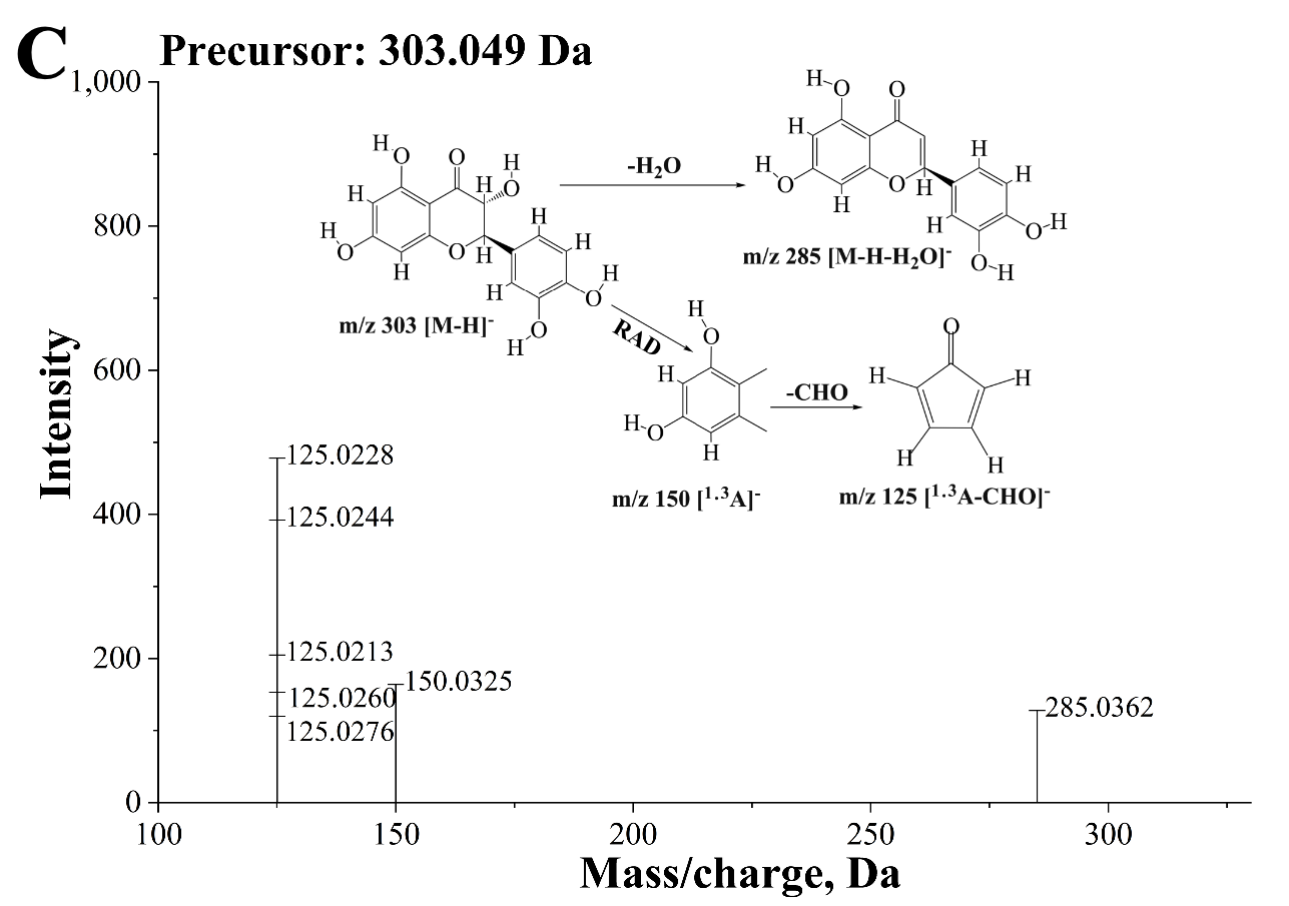

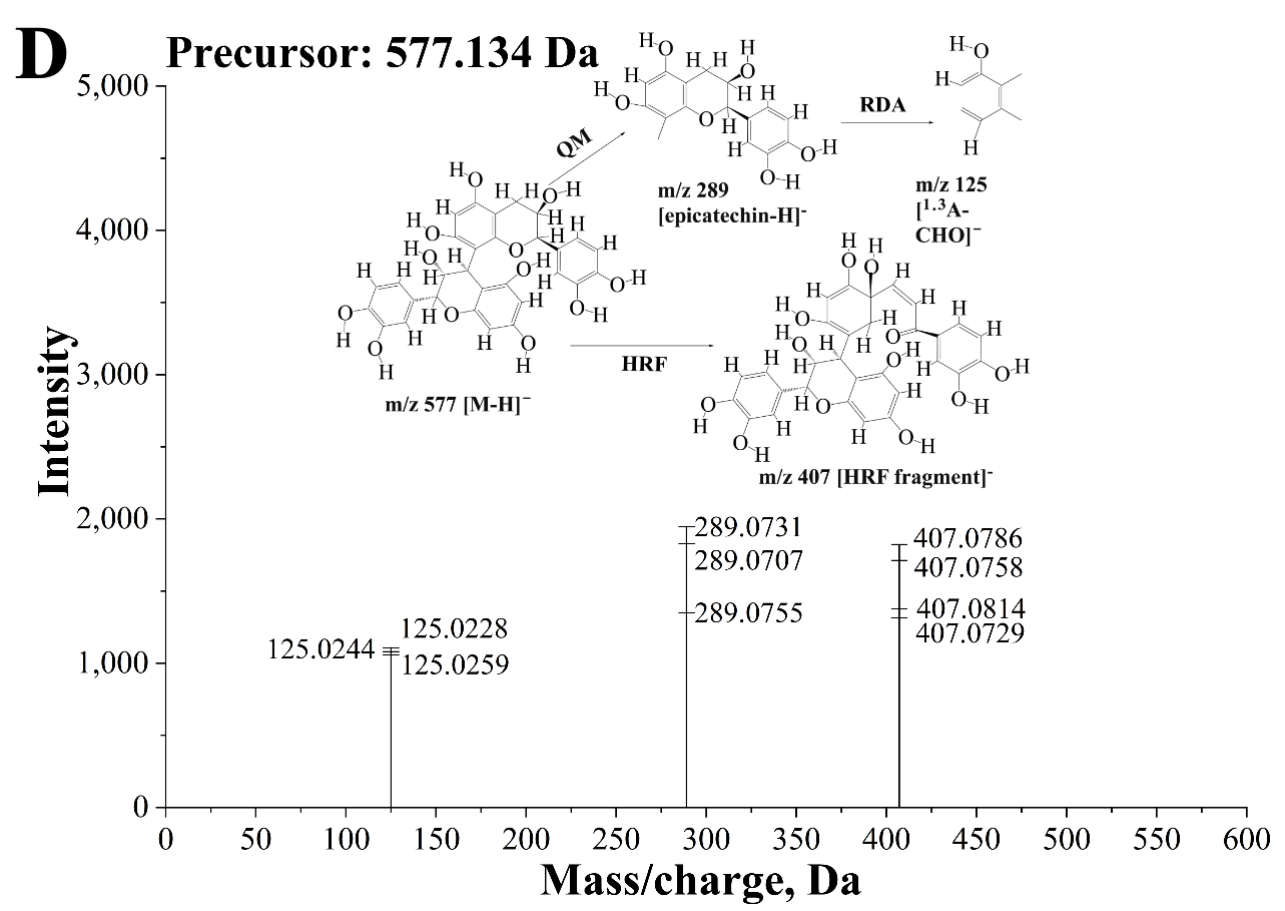
MS/MS spectra and proposed fragmentation pathways of representative flavonoid compounds in the OT sample, exemplified by (**A**) quercitrin, (**B**) tiliroside, (**C**) taxifolin, and (**D**) procyanidin B2.

In this study, non-targeted metabolomics analysis of flavonoids in PN extracts was performed using UPLC-Triple-TOF/MS technology combined with an Information-Dependent Acquisition (IDA) strategy. Peak extraction, alignment, and database matching (Metlin, MassBank, MoNA, HMDB) were carried out using MS-DIAL software (ver 4.6). The identification of flavonoid components relied on accurate mass measurements, retention times, and MS/MS fragmentation profiles. The fragmentation pathways of the flavonoids were analyzed according to the MS/MS spectra (using quercitrin, tiliroside, taxifolin, and procyanidin B2 from the OT sample as examples), as shown in Figure 6.

Quercitrin, a representative flavonol-3-O-glycoside in pine needles, consists of a quercetin aglycone linked to a rhamnose moiety at the C3 position; its deprotonated molecular ion [M-H]^−^ appeared at *m*/*z* 447.09489 (C_21_H_20_O_11_), and the MS/MS spectrum (Figure 6A) exhibited characteristic fragment ions at *m*/*z* 284.03047, 285.03886, 255.02874, and 227.03413. In the negative ion mode, the C3-O-rhamnosidic bond of quercitrin underwent heterolytic cleavage, resulting in the loss of a rhamnosyl group (146 Da) and generating the quercetin aglycone ion [quercetin-H]^−^, corresponding to the fragment ion at *m*/*z* 285.03886 (intensity 1250). This ion serves as a characteristic diagnostic ion for flavonol-3-O-glycosides. Concurrently, homolytic cleavage of the glycosidic bond occurred, leading to the loss of a rhamnosyl radical and producing the quercetin aglycone radical anion [quercetin-H]^−^•, corresponding to the fragment ion at *m*/*z* 284.03047 (intensity 3839). The intensity of this radical anion was significantly higher than that of the aglycone ion, representing a characteristic fragmentation behavior of flavonol-3-O-glycosides in the negative ion mode, and indicating that homolytic cleavage was the predominant fragmentation pathway. The aglycone radical anion at *m*/*z* 284 further lost one molecule of carbon monoxide (CO, 28 Da), generating the fragment ion at *m*/*z* 255.02874 (intensity 3706), which is a characteristic product of C-ring cleavage. Subsequently, the aglycone ion at *m*/*z* 285 underwent retro-Diels–Alder (RDA) fragmentation, with the C-ring cleaved at the C1-C2 and C3-C4 bonds, producing the A-ring characteristic fragment ion at *m*/*z* 227.03413 (intensity 2375). This fragment corresponds to [1.3A]^−^ and is characteristic of an A-ring with 5,7-dihydroxy substitution. In this study, the intensity of *m*/*z* 284 (3839) was significantly higher than that of *m*/*z* 285 (1250) [52], and the presence of the low-mass fragment ion *m*/*z* 227 further confirmed the 5,7-dihydroxy substitution pattern of the A-ring [51]. The observed fragmentation pattern is essentially consistent with the characteristic fragmentation behavior of flavonol-3-O-glycosides in the negative ion mode [53,54,55].

Tiliroside, a representative flavonol acylglycoside in pinus needles, is composed of a kaempferol aglycone linked to a 6″-O-p-coumaroylglucosyl moiety at the C3 position [56]. The [M-H]^−^ ion of tiliroside was observed at *m*/*z* 593.13053 (C_30_H_26_O_13_), and its MS/MS spectrum (Figure 6B) displayed characteristic fragment ions at *m*/*z* 284.03208 and 285.04046. In the negative ion mode, tiliroside initially underwent glycosidic bond cleavage, with the sequential loss of a p-coumaroyl group (146 Da) and a glucosyl group (162 Da), resulting in a total loss of 308 Da and generating the kaempferol aglycone ion [kaempferol-H]^−^, corresponding to the fragment ion at *m*/*z* 285.04046 (intensity 5504). This fragment serves as a characteristic diagnostic ion for flavonol glycosides. Concurrently, homolytic cleavage of the glycosidic bond released a glycosyl radical, generating the kaempferol aglycone radical anion [kaempferol-H]^−^• at *m*/*z* 284.03208 (intensity 4916). The intensity of this radical anion was comparable to that of the aglycone ion, representing a characteristic fragmentation behavior of flavonol-3-O-glycosides in the negative ion mode, and indicating that homolytic and heterolytic cleavages occurred simultaneously. Notably, in this study, the intensity of *m*/*z* 284 (4916) was comparable to that of *m*/*z* 285 (5504), which is consistent with the characteristic feature of flavonol-3-O-glycosides undergoing both homolytic and heterolytic cleavages simultaneously in the negative ion mode [57,58].

Taxifolin (also known as dihydroquercetin) is a representative dihydroflavonol compound found in pine needles; it consists of a quercetin skeleton with a saturated C-ring, in which the C2-C3 bond is a single bond and a hydroxyl group is attached to the C3 position [40]. The [M-H]^−^ ion of taxifolin was observed at *m*/*z* 303.049 (C_15_H_11_O_7_), and its MS/MS spectrum (Figure 6C) displayed characteristic fragment ions at *m*/*z* 285.03616, 150.03253, and 125.02284. In the negative ion mode, taxifolin underwent dehydration at the C3 hydroxyl group, with the loss of one molecule of water (18 Da), generating the dehydrated fragment ion [M-H-H_2_O]^−^, which corresponds to the fragment ion at *m*/*z* 285.03616 (intensity 128). This fragment serves as a characteristic diagnostic ion for dihydroflavonol compounds, corresponding to a flavonoid structure with a double bond formed between the C2 and C3 positions of the C-ring. Simultaneously, RDA fragmentation occurred, with the C-ring cleaved at the C1-C2 and C3-C4 bonds, producing the A-ring characteristic fragment [1.3A]^−^, which corresponds to the fragment ion at *m*/*z* 150.03253 (intensity 164). This fragment is characteristic of an A-ring with a 5,7-dihydroxy substitution. The A-ring characteristic fragment at *m*/*z* 150 further lost one molecule of CHO (29 Da), generating the fragment ion at *m*/*z* 125.02284 (intensity 478). This fragment corresponds to [1.3A-CHO]^−^ and is a characteristic product of further A-ring cleavage [52,59,60].

Proanthocyanidins are important polyphenolic constituents of pine needles, and their mass spectrometric fragmentation patterns are highly characteristic. Procyanidin B2, a representative B-type proanthocyanidin dimer in PN, is composed of two (-)-epicatechin units linked via a C4→C8 interflavan bond [61]. The MS/MS spectrum of procyanidin B2 clearly illustrates the typical fragmentation pathway of B-type proanthocyanidin dimers (Figure 6D); its deprotonated molecular ion [M-H]^−^ appears at *m*/*z* 577.1345 (C_30_H_25_O_12_^−^), and the MS/MS spectrum exhibits characteristic fragment ions at *m*/*z* 289.07308, 407.07858, and 125.02435. In negative ion mode, cleavage of the C4-C8 bond in procyanidin B2 generated the (-)-epicatechin monomer unit [epicatechin-H]^−^ at *m*/*z* 289.07308 (intensity 1947). This fragment, which served as the base peak, is a diagnostic ion for B-type proanthocyanidin dimers. Concurrent heterocyclic ring fission (HRF) occurred, with cleavage at the C1-C2 and C4-C4a bonds, yielding a dimeric fragment that retained the C4-C8 linkage at *m*/*z* 407.07858 (intensity 1823). This fragment, differing from the parent ion by 170 Da, represents another characteristic ion of B-type proanthocyanidin dimers [62,63]. Further retro-Diels–Alder (RDA) fragmentation of the monomeric fragment at *m*/*z* 289 occurred via cleavage of the C-ring at the C1-C2 and C3-C4 bonds, generating the A-ring fragment at *m*/*z* 125.02435 (intensity 1080), which is characteristic of a 5,7-dihydroxy substitution pattern on the A-ring [64]. Notably, the extra C2-O-C7 ether bond in A-type proanthocyanidin dimers results in a quinone methide (QM) cleavage product at *m*/*z* 287, which is 2 Da lower than that of B-type dimers. The absence of an *m*/*z* 287 ion further confirms procyanidin B2 as a B-type proanthocyanidin dimer [65]. The intensity of the QM cleavage product at *m*/*z* 289 (1947) was markedly higher than that of the HRF product at *m*/*z* 407 (1823), indicating that QM cleavage is the predominant fragmentation pathway for procyanidin B2 in negative ion mode [66]. The observed fragmentation behavior is fully consistent with previously reported patterns for B-type proanthocyanidin dimers [67], and the presence of the A-ring fragment at *m*/*z* 125 further corroborates the 5,7-dihydroxy substitution pattern.

#### 3.4.2. Analysis of Compositional Differences in Flavonoids Extracted from PN via Two Methods

**Figure 7:**
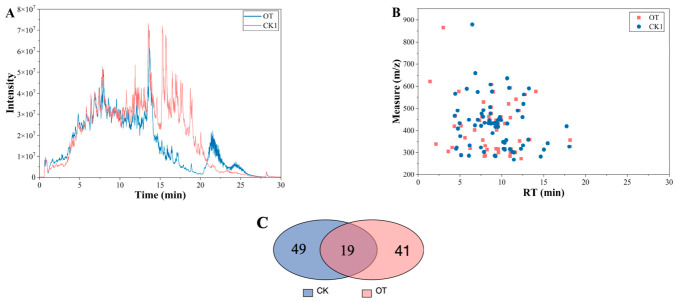
Untargeted global characterization of flavonoids extracted from PN via two different methods: (**A**) TIC chromatograms in negative ion mode. (**B**) Two-dimensional scatterplot of retention time versus *m*/*z*. (**C**) Venn diagram showing common and unique flavonoid compounds between the two groups.

Figure 7A depicts the total ion current (TIC) chromatograms of the flavonoid extracts obtained under the two extraction protocols. A comprehensive analysis of the chromatographic profiles led to the identification of 110 flavonoid compounds in total (Tables S1 and S2, Supplementary Materials). The corresponding retention time–*m*/*z* relationships are visualized as a two-dimensional scatterplot in Figure 7B. All flavonoids were eluted within 20 min, with the majority eluting between 5 and 15 min. Among them, 60 compounds were identified in the OT group and 68 compounds in the CK group. The compositional differences in the flavonoids extracted from PN via different methods are shown in Figure 7C. Comparative analysis revealed 19 identical compounds between the two extracts, representing the core flavonoid constituents of PN [19], including kaempferol, myricetin, quercitrin, kaempferol-3-O-α-L-rhamnoside, and (-)-epicatechin.

**Figure 8:**
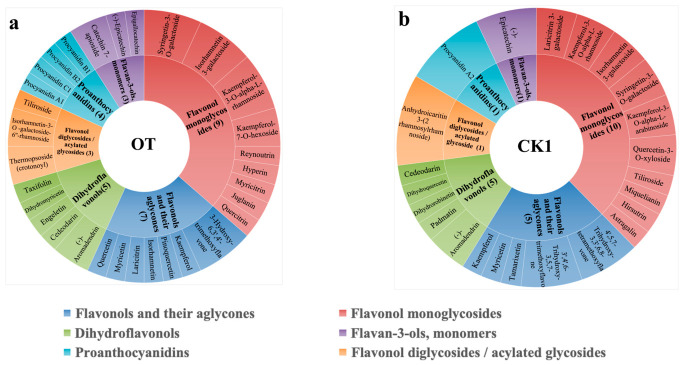
Comparative classification of the major flavonoid components extracted from PN via two different methods. (**a**) Major flavonoid components identified in the purified extract obtained under the optimal enzyme-assisted ultrasonic extraction conditions; (**b**) Major flavonoid components identified in the flavonoids extracted and purified by the conventional method.

Marked differences in the distribution of flavonoid subclasses were evident between the two extracts, as shown in Figure 8. The compositional variations primarily involved three aspects: the ratio of aglycones to glycosides, the release of proanthocyanidin oligomers, and the diversity of glycosides. In the OT group, seven flavonols and their aglycones were identified, with unique compounds including quercetin, isorhamnetin, and pinoquercetin; these aglycones possess stronger free radical scavenging capacity and higher bioavailability. In contrast, only five such compounds were identified in the CK group, indicating that enzymatic hydrolysis effectively promoted the hydrolysis of flavonoid glycosides. Meanwhile, the OT group exhibited a pronounced enrichment advantage in flavan-3-ols and proanthocyanidins. Three flavan-3-ol monomers (epigallocatechin, catechin 7-apioside, and (-)-epicatechin) and four proanthocyanidins (procyanidins B1, B2, C1, and A1) were identified in the OT group. In contrast, only one flavan-3-ol monomer ((-)-epicatechin) and one proanthocyanidin (procyanidin A2) were identified in the CK group. Proanthocyanidins are important components of bound polyphenols in the plant cell wall and are typically associated with cell-wall polysaccharides such as cellulose and hemicellulose via hydrogen bonds or covalent bonds. The synergistic action of cellulase and pectinase effectively degraded the polysaccharide skeleton of the cell wall, releasing bound proanthocyanidin oligomers [61]. In this study, the detection of procyanidins B1, B2 (dimers), C1 (trimer), and A1 (A-type dimer) in the OT group formed a complete series of proanthocyanidins, confirming that the enzyme-assisted ultrasonic method has advantages in releasing high-molecular-weight flavonoids. Three flavonol diglycosides/acylglycosides (isorhamnetin-3-O-galactoside-6″-rhamnoside, thermopsoside, and tiliroside) were identified in the OT group, whereas only one such compound (anhydroicaritin 3-(2 rhamnosylrhamnoside)) was identified in the CK group. Among these, tiliroside is kaempferol-3-O-p-coumaroylglucoside, which has well-documented anti-inflammatory, antioxidant, and anti-glycation activities and is an important bioactive component of pine needle flavonoids. The detection of this compound further demonstrates the advantage of the enzyme-assisted ultrasonic method in extracting flavonoids with complex structures [56]. In summary, the synergistic extraction method combining enzymatic hydrolysis and ultrasonication is more suitable for enriching highly active flavonoid components.

### 3.5. FTIR and DSC Analysis of Flavonoid Compounds from PN

**Figure 9:**
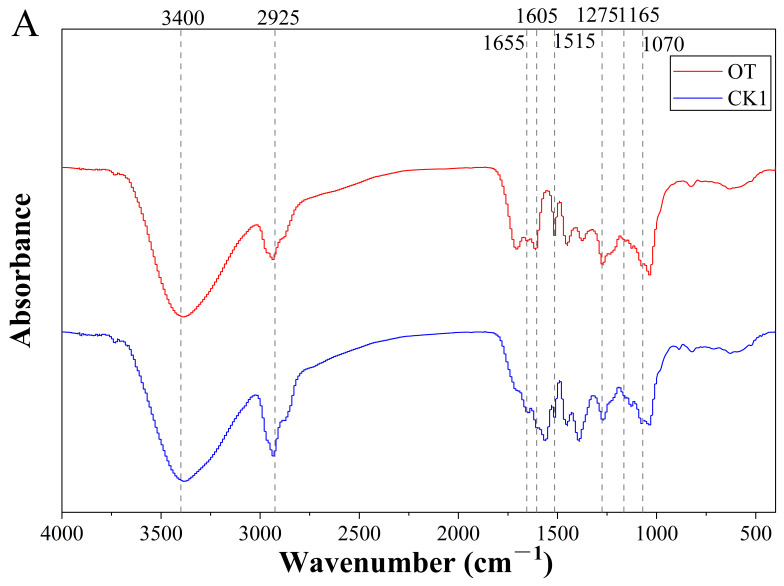

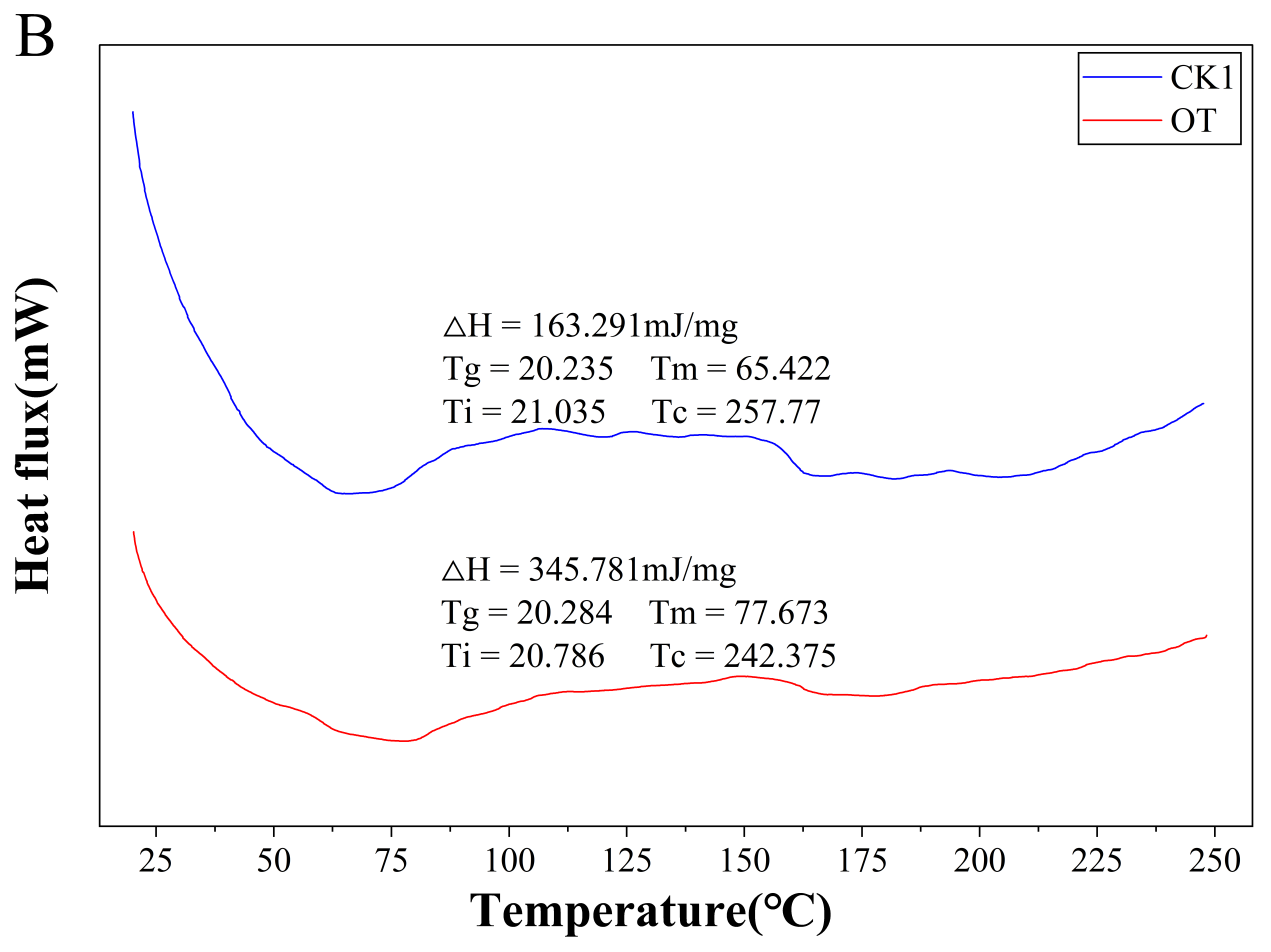
FTIR spectra (**A**) and DSC curves (**B**) of flavonoids from PN.

The structural characteristics of the flavonoid compounds extracted from PN in the OT and CK1 groups were analyzed by FTIR spectroscopy. As shown in Figure 9A, both samples exhibited characteristic absorption bands of flavonoids at 3400 cm^−1^, 2925 cm^−1^, 1655 cm^−1^, 1605 cm^−1^, 1515 cm^−1^, 1275 cm^−1^, 1165 cm^−1^, and 1070 cm^−1^. Specifically, the broad absorption band around 3400 cm^−1^ was assigned to O-H stretching vibrations (phenolic and alcoholic hydroxyl groups), the absorption band at 1655 cm^−1^ was attributed to the C=O stretching vibration of the C-ring carbonyl group in flavonoids, the bands at 1605 cm^−1^ and 1515 cm^−1^ corresponded to aromatic C=C skeleton vibrations, and the strong absorption in the 1200–1000 cm^−1^ region was assigned to C-O-C stretching vibrations of glycosidic bonds [68,69]. The OT group exhibited a stronger O-H absorption band at 3400 cm^−1^, which is consistent with its enrichment of flavonol aglycones. Flavonol aglycones (e.g., quercetin and kaempferol) possess free phenolic hydroxyl groups at the C3 and C5 positions, leading to stronger O-H stretching vibrations in the FTIR spectrum [70]. Furthermore, the OT group exhibited stronger C-O-C absorption peaks in the region of 1200–1000 cm^−1^, indicating that it contained a higher abundance of flavonoid glycosides. This observation is consistent with the mechanism by which EAU promotes the release of bound glycosides from the cell wall [71]. Comparison of the FTIR spectra of the two sample groups revealed that the absorption peak intensities of the OT group in the regions of 3400 cm^−1^, 1655 cm^−1^, 1605 cm^−1^, and 1200–1000 cm^−1^ were all higher than those of the CK1 group. This suggests that the OT extract not only had a higher *TFC* but also contained more free phenolic hydroxyl groups and glycosidic bonds [72]. These findings are in good agreement with the UPLC-Triple-TOF/MS results, which demonstrated that the OT group was enriched in a greater variety of flavonol aglycones and flavonoid glycosides.

The DSC thermograms of CK1 and OT are shown in Figure 9B, exhibiting glass transition temperatures (Tg) of approximately 20.6 °C for CK1 and 20.5 °C for OT, indicating similar backbone structures [73,74]. The melting peak temperature (Tm) of OT (77.673 °C) was 12.3 °C higher than that of CK1 (65.422 °C), while the melting enthalpy of OT (ΔH = 345.781 mJ/mg) was significantly higher than that of CK1 (163.291 mJ/mg), representing an increase of approximately 112%. These results indicate that OT possesses a more ordered crystalline structure and higher crystallinity, which may be attributable to the enrichment of flavonoid aglycones or specific flavonoid components with stronger crystallization ability by the enzyme–ultrasonic synergistic extraction [44,75]. Regarding thermal stability, CK1 exhibited a higher decomposition temperature (Tc = 257.777 °C) than OT (242.375 °C), with a decrease of approximately 15.4 °C for OT. The reduced thermal stability of OT may be attributable to partial deglycosylation induced by the enzyme–ultrasonic treatment, which exposes more phenolic hydroxyl groups susceptible to thermal oxidation, as well as potential pre-oxidation of flavonoid molecules caused by ultrasonic cavitation [76,77].

### 3.6. In Vitro Bioactivity Evaluation of Flavonoids from PN

#### 3.6.1. In Vitro Antioxidant Activity Analysis

Previous studies have confirmed that pine needle extracts possess excellent in vitro antioxidant properties. The *TFC* values of *Pinus pinaster* Aiton (PPN) and *Pinus halepensis* Mill (PAN) needle extracts were 109.44 ± 0.62 and 111.64 ± 0.62 mg QE/g DM, respectively, and both extracts exhibited significant in vitro antioxidant activity. Specifically, the DPPH radical scavenging IC_50_ values were 0.47 ± 0.21 μg/mL for PPN and 1.49 ± 0.01 μg/mL for PAN, while the ABTS radical scavenging IC_50_ values were 2.69 ± 0.06 mg/mL for PPN and 10.9 ± 2.1 mg/mL for PAN. The total antioxidant capacity of the PAN needle extract reached 938.50 ± 23.23 mg AAE/g DM, while that of PPN was 182.01 ± 10.10 mg AAE/g DM [22]. Needle extracts of *Pinus mugo* Turra collected from three different mountainous regions in Romania exhibited *TFC* values ranging from 26.89 ± 0.2 to 45.55 ± 0.2 mg QE/g DM, and all showed good DPPH radical scavenging activity, with IC_50_ values ranging from 10.7 ± 0.12 to 13.2 ± 0.23 μg/mL [5]. The extract of *Pinus elliottii* needles also demonstrated significant in vitro antioxidant capacity, with a DPPH radical scavenging IC_50_ value of 41.05 ± 0.48 μg/mL, which was close to that of the positive control vitamin C (IC_50_ = 33.41 ± 0.12 μg/mL). The FRAP value was 1.09 ± 0.01 mM Fe^2+^/g PN, and the ABTS radical scavenging IC_50_ value was 214.07 ± 0.26 μg/mL [44]. Collectively, these studies confirm that needle extracts from different pine species exhibit good in vitro antioxidant activity, although their potency is influenced by factors such as species and geographical origin.

**Figure 10:**
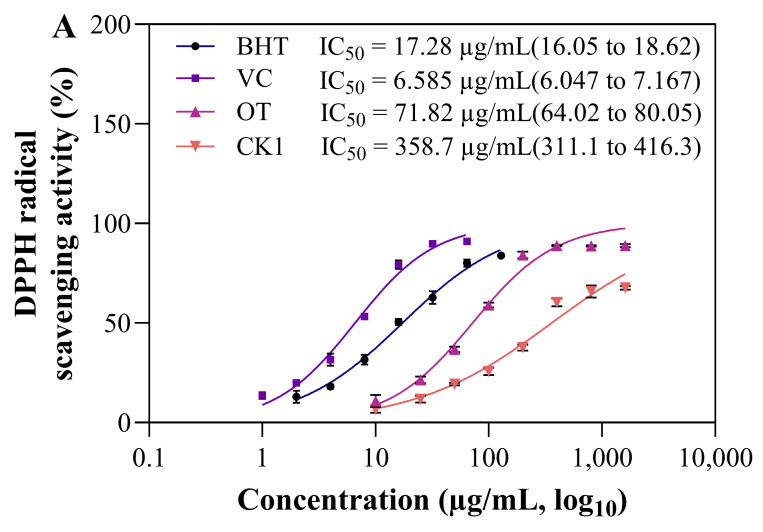

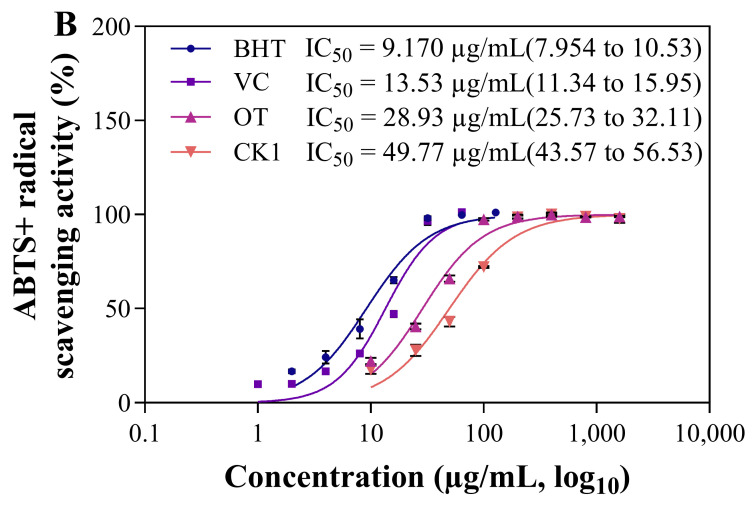

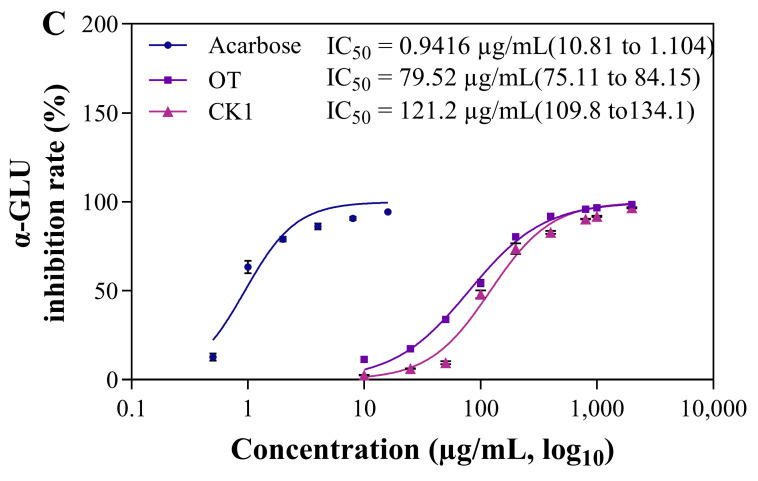

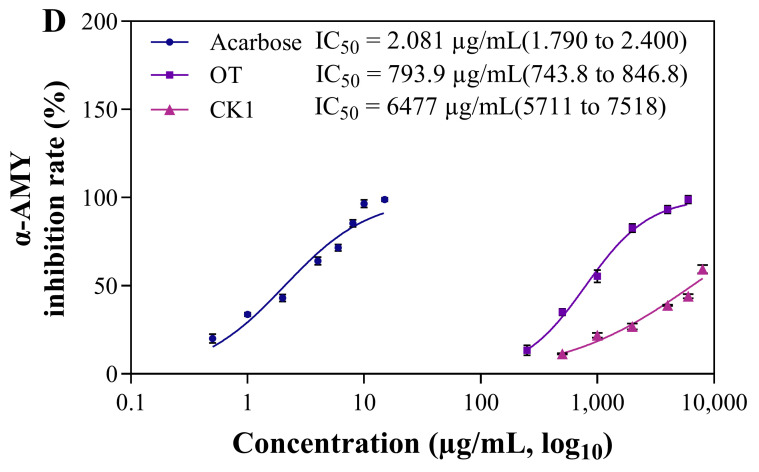
In vitro bioactivity analysis of flavonoids from PN, including (**A**) DPPH and (**B**) ABTS cationic radical scavenging activities, as well as (**C**) α-GLU- and (**D**) α-AMY-inhibitory activities. VC was used as a positive control in the antioxidant assays. BHT was used as a lipophilic antioxidant positive control. Acarbose was used as a positive control for the α-GLU and α-AMY inhibition assays.

The in vitro DPPH and ABTS^+^ radical scavenging activities of CK1, OT, BHT, and VC were assessed, and the IC_50_ values were derived from nonlinear regression analysis (Figure 10A,B). The radical scavenging rate of all samples increased with increasing concentration, showing a clear dose–response relationship [45]. As shown in Figure 10A, VC demonstrated the best DPPH radical scavenging activity, recording an IC_50_ value of 6.585 µg/mL, followed by BHT (IC_50_ = 17.28 µg/mL). Among the two flavonoid extracts, OT showed a significantly lower IC_50_ value (71.82 µg/mL), approximately one-fifth that of CK1 (358.7 µg/mL), indicating that the flavonoids in the OT group possessed stronger in vitro DPPH radical scavenging activity. At a concentration of 200 µg/mL, the DPPH radical scavenging rate of OT reached 84.2%. At higher concentrations (400–1600 µg/mL), the scavenging rate of OT stabilized at approximately 88–89%, indicating that its antioxidant activity reached a plateau within this concentration range. As shown in Figure 10B, BHT exhibited the strongest in vitro ABTS^+^ radical scavenging activity (IC_50_ = 9.170 µg/mL), followed by VC (IC_50_ = 13.53 µg/mL), OT (IC_50_ = 28.93 µg/mL), and CK1 (IC_50_ = 49.77 µg/mL). At a concentration of 100 µg/mL, the scavenging rate of OT reached 97.5%; at 50 µg/mL, the scavenging rate of OT (65.8%) was significantly higher than that of CK1 (43.1%). At higher concentrations (200–1600 µg/mL), the scavenging rate of OT stabilized at approximately 98–100%, reaching a plateau phase. In contrast, CK1 required higher concentrations to achieve a scavenging effect comparable to that of OT. These results indicate that, compared with the CE, the flavonoids obtained by the EAU exhibited significantly enhanced antioxidant activity.

Our findings are consistent with those of Li et al. [47] in the extraction of flavonoids from corn husk. Compared with conventional organic solvent extraction, cellulase–ultrasonic synergistic treatment significantly increased the *TFC* from 7.01 to 7.74 mg RE/g dw, and the DPPH and ABTS^+^ radical scavenging capacities of the synergistic extract were significantly higher than those of the conventional solvent extract. The enhanced in vitro antioxidant capacity of flavonoids extracted via EAU may be attributed to the partial deglycosylation of flavonoid glycosides induced by the EAU, which released aglycones with more free phenolic hydroxyl groups—a key structural feature for radical scavenging activity [75,78]. This result was validated by the previous component analysis. Additionally, ultrasonic cavitation may have induced structural rearrangement of the flavonoid molecules, thereby enhancing their electron-donating capacity [79,80,81]. Therefore, the EAU method not only improves the extraction yield of flavonoids but also significantly potentiates the in vitro antioxidant capacity of the extracts through the enrichment of additional synergistic components. Nonetheless, the in vivo antioxidant effects warrant further investigation via cellular or animal model studies.

#### 3.6.2. In Vitro α-Amylase- and α-Glucosidase-Inhibitory Activity Analysis

α-AMY and α-GLU, as key carbohydrate-digesting enzymes, are major targets for inhibiting postprandial blood glucose elevation and assisting in the prevention and management of diabetes mellitus [82]. Numerous studies have confirmed that natural products—particularly flavonoids—are rich sources of small-molecule inhibitors [83]; therefore, this study investigated the inhibitory effects of flavonoids extracted from PN on α-AMY and α-GLU. As shown in Figure 10C,D, the extracts exhibited concentration-dependent inhibitory activities against both α-AMY and α-GLU. The positive control acarbose showed the strongest inhibitory activity, with IC_50_ values of 2.081 µg/mL for α-AMY and 0.9416 µg/mL for α-GLU. The IC_50_ values of the OT group for α-AMY (793.9 µg/mL) and α-GLU (79.52 µg/mL) were significantly lower than those of the CK1 group (6477 µg/mL and 121.2 µg/mL, respectively), indicating stronger inhibitory activity. As shown in Figure 10C, at a concentration of 100 µg/mL, the α-GLU inhibition rate of OT reached 54.5%, while that of CK1 was only 48.0%. At 200 µg/mL, the inhibition rate of OT reached 80.3%, compared to 73.7% for CK1. At higher concentrations (400–2000 µg/mL), the inhibition rates of both OT and CK1 gradually approached that of acarbose, with OT reaching 98.6% at 2000 µg/mL. As shown in Figure 10D, at a concentration of 0.5 mg/mL, the α-AMY inhibition rate of the OT group reached 39.4%, while that of the CK1 group was only 14.0% under the same concentration. At 2 mg/mL, the inhibition rate of OT reached 79.4%, compared to only 27.9% for CK1. At 4 mg/mL, the inhibition rate of OT reached 93.2%. In contrast, CK1 required higher concentrations to achieve a comparable inhibitory effect to that of OT.

Lim et al. [84] systematically investigated the inhibitory activities of 14 structurally diverse flavonoids against human pancreatic α-AMY and two human intestinal α-GLU subunits (Nt-MGAM and Ct-MGAM), revealing the structural basis of flavonoids as starch-digesting enzyme inhibitors. Among these, luteolin and quercetin exhibited strong inhibitory activities against α-AMY, with maximum inhibition rates of 91.28% and 90.18%, and IC_50_ values of 34.83 μM and 35.21 μM, respectively. Additionally, quercetin showed potent inhibitory activity against Nt-MGAM and Ct-MGAM, with IC_50_ values of 28.75 μM and 31.46 μM, respectively, and the inhibition type was competitive. Molecular docking analysis revealed that these two flavonoids formed hydrogen bonds with key amino acid residues in the catalytic active site of α-AMY, such as Arg195, Glu233, and Gln63, through the C2=C3 double bond and hydroxyl groups at positions A5, B3, and B4, allowing them to bind parallel to the wide and shallow catalytic groove on the enzyme surface. Furthermore, the hydroxyl groups at B3, B4, and C3 of quercetin formed hydrogen bonds with residues such as Asp327, Asp443, and Asp542 in the catalytic active site, enabling B-ring-specific insertion into the narrow and deep catalytic pocket of the enzyme. These findings confirm that flavonoids are effective small-molecule inhibitors of α-AMY and α-GLU, and that selective inhibition of these two enzymes can be achieved through specific hydroxyl substitution patterns and C-ring structures. Laaraj et al. [85] found that the acetone extract of pomegranate (*Punica granatum*) bark exhibited 95.85% inhibition against α-GLU at a concentration of 166 μg/mL, with an IC_50_ value of 96.19 μg/mL, and an IC_50_ value of 373.90 μg/mL against α-AMY. HPLC-DAD analysis identified several flavonoids, including (−)-catechin, rutin, and naringenin. Correlation analysis revealed that *TFC* was significantly positively correlated with α-AMY-inhibitory activity, and (−)-catechin showed an IC_50_ value of 23.42 μg/mL against α-AMY. Molecular docking further confirmed that these compounds exert competitive inhibition by forming hydrogen bonds with the enzyme’s active site. Lim et al. [86] reported that the New Zealand pine bark (*Pinus radiata*) extract contained over 80% proanthocyanidins of varying degrees of polymerization. This extract exhibited potent enzyme-inhibitory activities, with IC_50_ values of 3.98 ± 0.11 mg/mL against α-AMY and 13.02 ± 0.28 μg/mL against α-GLU, the latter being approximately 306 times more potent than the former. The mechanism of action is closely related to the polyphenolic hydroxyl structure and the degree of polymerization of the flavonoids. Oligomeric proanthocyanidins exert competitive inhibition by specifically interacting with the active binding cavity of the enzyme protein, while higher-molecular-weight proanthocyanidins, due to their lower bioavailability, may accumulate in the small intestine and inhibit enzyme activity by interacting with the enzyme surface, leading to protein aggregation and precipitation. The authors compared the IC_50_ values of French maritime pine bark extract and Korean pine bark extract against α-GLU (5.34 μg/mL and 0.025 μg/mL, respectively), and they concluded that differences in IC_50_ values among studies may arise from variations in extract composition, purification methods, and assay conditions (e.g., pH, reaction time, temperature, substrate concentration and source, enzyme concentration and source).

In the present study, the OT group was identified by UPLC-Triple-TOF/MS to contain flavonoid compounds including luteolin, quercetin, catechin-7-glucoside, taxifolin, and four proanthocyanidins (proanthocyanidin B1, B2, C1, and A1), whereas these compounds were not identified in the CK1 group (Table S1). Collectively, these findings offer a molecular-level interpretation of the potent α-AMY- and α-GLU-inhibitory activities exhibited by the OT group, as well as suggesting that the overall activity of the plant extract arises from the synergistic actions of multiple constituents. However, which specific flavonoid compounds contributed to the enhanced bioactivity in this study remains unclear. Further research, such as fractionation, bioassay-guided isolation, or molecular docking, will be needed to identify the key bioactive components. Sun et al. [87] optimized the ultrasound-assisted extraction process of polyphenols from *Paulownia flowers* using RSM, and they identified 32 compounds by HPLC-MS/MS, with luteolin (0.46 μg/g) and apigenin (0.32 μg/g) as the major flavonoid components. The purified product exhibited mixed-type inhibition against α-GLU, confirming that it forms a complex with the enzyme through a static mechanism, inducing conformational changes in the secondary structure of the enzyme. Molecular docking revealed that luteolin and apigenin competitively bind to the enzyme’s active site through hydrogen bonding and hydrophobic interactions. These findings demonstrate that optimized extraction and purification methods can effectively enrich flavonoid compounds, thereby significantly enhancing their inhibitory activity against α-GLU. The present study’s findings demonstrate that the extraction method directly affects the yield of flavonoids and their enzyme-inhibitory activity. Compared with the CE, the EAU resulted in an 8.2-fold increase in α-AMY-inhibitory activity. This enhancement may be attributed to the structural modifications induced by the treatment, such as partial deglycosylation and the exposure of specific phenolic hydroxyl groups, which may be more favorable for interaction with the active site of α-AMY [88,89]. The above findings highlight the in vitro inhibitory activity of the flavonoid compounds against α-GLU and α-AMY, suggesting their potential for modulating postprandial blood glucose. This provides preliminary evidence for flavonoids as dual inhibitors of carbohydrate-digesting enzymes.

## 4. Conclusions

In this study, single-factor experiments combined with RSM were employed to determine the optimal process parameters for EAU of flavonoids from PN. The optimized extraction process significantly improved the *TFC*, reaching 17.08 mg rutin/g dry matter in the EAU group. SEM revealed a marked structural alteration in the needle residues of the EAU group, indicating that the combined EAU exerted an erosive and penetrating effect on the pine needle tissue. Through UPLC-Triple-TOF/MS analysis, the major components of the flavonoids from PN were preliminarily identified. The optimized extraction process exhibited enrichment advantages for flavonols and their aglycones, flavan-3-ols, proanthocyanidins, and flavonol diglycosides/acylglycosides. The main identified compounds included quercitrin, quercetin, isorhamnetin, pinoquercetin, kaempferol, myricetin, kaempferol-3-O-α-L-rhamnoside, (-)-epicatechin, thermopsoside, and tiliroside. FTIR and DSC structural analyses indicated that the flavonoids in the OT group possessed higher crystallinity and a more ordered crystalline structure, although their thermal stability was slightly reduced, which may be attributable to deglycosylation and structural changes induced by ultrasonic cavitation. By determining the IC_50_ values for DPPH and ABTS^+^ radical scavenging, as well as for α-GLU and α-AMY inhibition, the OT group exhibited superior in vitro antioxidant activity and in vitro carbohydrate-digesting-enzyme-inhibitory activity. This study provides a new approach for the valorization of pine needle forestry waste and offers a preliminary experimental basis for the potential application of pine needle flavonoids in functional foods and related fields. It should be noted, however, that all bioactivity assessments in this study were based on in vitro biochemical assays, and no cellular or animal model validation was performed. Therefore, the physiological efficacy, safety, and practical application potential of the flavonoids from pine needles require further investigation.

## Figures and Tables

**Table 1:** Mobile phase elution gradient.

Time (min)	Flow Rate (μL/min)	A (%)	B (%)
0.00	400	95	5
1.50	400	95	5
2.50	400	90	10
14.00	400	60	40
24.00	400	5	95
27.00	400	5	95
27.10	400	95	5

**Table 2:** Variables and levels of BBD.

Symbols	Independent Variables	Factor Levels		
		−1	0	1
A	Enzyme dosage (wt%)	1.5	2.0	2.5
B	Ethanol concentration (%)	60	70	80
C	Ultrasonication time (min)	15	20	25

**Table 3:** Three level Box–Behnken experimental design for flavonoid compound extraction (*n* = 3).

Run	A	B	C	Response Value
	Enzyme Dosage (wt%)	Ethanol Concentration (%)	Ultrasonication Time (min)	*TFC* (mg Rutin/g Dry Matter)
1	2.0	80	15	13.3314
2	2.0	70	20	15.9471
3	2.0	70	20	16.8514
4	2.0	60	25	12.621
5	2.5	70	25	14.1755
6	2.0	70	20	16.4466
7	2.0	70	20	16.2421
8	2.5	70	15	14.042
9	2.0	70	20	16.3679
10	2.0	60	15	13.0048
11	1.5	70	15	15.2471
12	1.5	70	25	15.7042
13	2.5	80	20	13.9913
14	1.5	60	20	13.8528
15	2.5	60	20	13.2181
16	1.5	80	20	14.9471
17	2.0	80	25	14.3017

**Table 4:** ANOVA for the quadratic polynomial model.

Source	Sum of Squares	df	Mean Square	*F*-Value	*p*-Value	
Model	28.51	9	3.17	37.04	<0.0001 ^b^	Significant
*A*	2.34	1	2.34	27.33	0.0012 ^a^	
*B*	1.88	1	1.88	21.95	0.0022 ^a^	
*C*	0.1732	1	0.1732	2.03	0.1977	
*AB*	0.0258	1	0.0258	0.3015	0.6000	
*AC*	0.0262	1	0.0262	0.3061	0.5973	
*BC*	0.4584	1	0.4584	5.36	0.0538	
*A^2^*	0.8360	1	0.8360	9.78	0.0167 ^a^	
*B^2^*	15.57	1	15.57	182.09	<0.0001 ^b^	
*C^2^*	5.41	1	5.41	63.22	<0.0001 ^b^	
Residual	0.5986	7	0.0855			
Lack of Fit	0.1659	3	0.0553	0.5110	0.6960	Not Significant
Pure Error	0.4328	4	0.1082			
Cor Total	29.11	16				
*R*^2^ = 0.9794; Adjusted *R*^2^ = 0.9530; Adeq Precision = 16.6395						

^a^ Significant at the 0.05 level; ^b^ significant at the 0.001 level.

## Data Availability

The original contributions presented in this study are included in the article/Supplementary Materials. Further inquiries can be directed to the corresponding author.

## Supplementary Materials

The following supporting information can be downloaded at https://www.mdpi.com/article/10.3390/antiox15060712/s1.

## Abbreviations

The following abbreviations are used in this manuscript:

PN	*Pinus koraiensis* needle
EAU	enzyme-assisted ultrasonic extraction
CE	conventional extraction
EE	enzyme-assisted ethanol extraction without ultrasonic treatment
UE	ultrasound-assisted ethanol extraction without enzyme pretreatment
RSM	Response surface methodology
*TFC*	Total flavonoid content
SEM	Scanning electron microscopy
UPLC-Triple-TOF/MS	Triple-quadrupole time-of-flight mass spectrometry
FTIR	Fourier-transform infrared spectroscopy
DSC	Differential scanning calorimetry
α-GLU	α-Glucosidase
α-AMY	α-Amylase
VC	L (+)-ascorbic acid
DMSO	Dimethyl sulfoxide
BHT	2,6-Di-tert-butyl-4-methylphenol
DPPH	2,2-Diphenyl-1-picrylhydrazyl
ABTS	2,2′-Azino-bis (3-ethylbenzothiazoline-6-sulfonic acid)
DNS	3,5-Dinitrosalicylic acid
OT	purified extract obtained under the optimal enzyme-assisted ultrasonic extraction conditions
CK1	purified flavonoids obtained by conventional extraction
