# Supplementary material for: Enzyme-Assisted Ultrasonic Extraction of Flavonoids from Pinus koraiensis Needle Litterfall: Process Optimization, Component Identification, and In Vitro Bioactivity Evaluation
Source: Antioxidants (Basel). 2026 Jun 3;15(6):712. doi: 10.3390/antiox15060712 (PMC13295667; doi:10.3390/antiox15060712)
Supplement: Supplementary file 1 [file antioxidants-15-00712-s001.zip › antioxidants-4329058-supplementary.pdf]

Table S1. Identification of flavonoids from purified extracts of PN in the OT group by UHPLC-MS/MS in negative ion mode ([M-H] ⁻). DP, FP, and TS represent dot product, fragment presence, and total score, respectively, which are applicable to the following tables as well.

No.	Title	RT (min)	Theoreti calm/z	Precursor m/z	PPM	Reference m/z	Formula	DP	FP (%)	TS	MS/MS Fragments m/z (Intensity)
1	isorhamnetin-3-O-galactoside-6''-rhamnoside	1.43	623.162	623.155	11.18	623.162	$C_{28}H_{32}O_{16}$	17.9	100	62.3	153.02972 (60); 467.13847 (48); 623.14686 (48); 623.15387 (48); 623.15737 (133); 623.16087 (73); 623.16438 (90); 623.16788 (65)
2	4',5,7-Trihydroxy-3'-prenylflavanone	2.13	339.124	339.126	7.37	339.124	$C_{20}H_{20}O_5$	12.3	83.3	62.2	58.00367 (24); 59.01264 (36); 59.0148 (60); 71.01513 (24); 85.02871 (24); 85.03 (24); 89.02383 (36); 89.02515 (24); 99.00613 (24); 113.02393 (24); 127.04883 (24); 161.04611 (24); 339.1274 (24);339.12999 (36)
3	Procyanidin C1	3.01	865.199	865.201	2.95	865.199	$C_{45}H_{38}O_{18}$	34.8	100	76.8	865.19627 (75); 865.2004 (82); 865.20452 (92); 865.20865 (74)
4	Epigallocatechin	3.61	305.067	305.065	4.19	305.067	$C_{15}H_{14}O_7$	80.3	71.4	86.7	125.02126 (81); 125.02283 (168); 125.0244 (270); 125.02597 (180); 125.02754 (86); 125.0291 (83); 137.02509 (105); 137.02673 (76)
5	Abyssinone II	4.07	323.129	323.132	8.71	323.129	$C_{20}H_{20}O_4$	21.2	50	61.2	59.0116 (297); 59.01268 (753); 59.01376 (619); 59.01484 (490); 59.01592 (291); 59.01699 (164); 71.01163 (118); 71.01281 (234); 71.014 (178); 85.02877 (113); 89.02256 (181); 89.02389 (2480; 89.02521 (166);n89.02786 (114);

											113.0255 (127); 119.03202 (137); 119.03355 (113); 119.03508 (264); 323.1307 (187); 323.13322 (197); 323.13574 (163); 323.13827 (122)
6	Pomiferin	4.33	419.150	419.156	13.33	419.150	$C_{25}H_{24}O_6$	13	100	60.5	419.15209 (152); 419.15496 (172)
7	Dichamanetin	4.44	467.150	467.149	3.03	467.150	$C_{29}H_{24}O_6$	14.2	66.7	60.8	153.05383 (540); 153.05557 (405); 153.05731 (305); 467.15609 (384)
8	Procyanidin B2	4.87	577.135	577.134	2.00	577.135	$C_{30}H_{26}O_{12}$	58	100	84.1	125.02278 (1108); 125.02435 (1080); 125.02592 (1059); 289.0707 (1828); 289.07308 (1947); 289.07547 (1350); 407.07292 (1314); 407.07575 (1713); 407.07858 (1823); 407.08141 (1379)
9	Reynoutrin	4.96	433.078	433.075	7.02	433.078	$C_{20}H_{18}O_{11}$	18.5	72	60.1	303.04074 (5187); 303.04318 (10552); 303.04563 (20126); 303.04807 (32088); 303.05051 (36558); 303.05296 (31902); 303.0554 (21939); 303.05784 (11999); 303.06028 (5518)
10	(-)-Epicatechin	5.13	289.072	289.072	1.51	289.072	$C_{15}H_{14}O_6$	71.3	88.5	86.5	109.02735 (90); 109.02882 (161); 109.03028 (126); 109.03321 (81); 123.04276 (188); 123.04432 (183); 123.04587 (134); 123.04743 (85); 125.02444 (65); 203.07094 (111); 245.07882 (69); 245.08541 (81); 289.06852 (85)
11	Catechin 7-apioside	5.13	421.114	421.112	5.04	421.114	$C_{20}H_{22}O_{10}$	13.2	100	68.9	361.09146 (408); 391.10573 (463); 421.11066 (346); 421.11354 (445)

12	Curcumin	5.61	367.119	367.123	10.86	367.119	$C_{21}H_{20}O_6$	11.1	83.3	62.7	85.0649 (403); 85.06619 (634); 85.06748 (248); 177.03698 (307); 177.03885 (329); 177.04072 (353); 177.04258 (269); 177.04445 (272); 367.12421 (245)
13	Dihydromyricetin	6.21	319.046	319.043	9.54	319.046	$C_{15}H_{12}O_8$	71.6	58.5	79.6	125.02126 (97); 125.02283 (145); 125.0244 (75); 125.02597 (173); 125.02754 (76); 191.03435 (97); 193.01198 (115); 193.01393 (236); 193.01588 (125)
14	Sericetin	6.75	403.155	403.157	3.72	403.155	$C_{25}H_{24}O_5$	12.4	100	64.9	121.02583 (316); 121.02737 (850); 121.02891 (814); 121.03046 (738); 121.032 (309); 403.15806 (270); 403.16088 (291)
15	Hovetrichoside C	7.12	449.109	449.110	1.92	449.109	$C_{21}H_{22}O_{11}$	30.3	82.4	68.7	151.00195 (440); 152.01068 (443); 152.01241 (518); 167.03463 (522); 269.0408 (439); 269.04311 (801); 269.04541 (1082); 269.04771 (710); 361.09452 (482); 449.10881 (479)
16	Baicalin	7.34	445.078	445.080	5.30	445.078	$C_{21}H_{18}O_{11}$	7.5	66.7	61.7	247.09725(894)
17	Myricitrin	7.62	463.088	463.087	3.46	463.088	$C_{21}H_{20}O_{12}$	43.8	100	79.6	316.01637 (431); 316.01886 (737); 316.02136 (1031); 316.02385 (1003); 316.02634 (602)
18	3-Hydroxy-7,8,2',3'-tetramethoxyflavone	7.73	357.098	357.098	1.18	357.098	$C_{19}H_{18}O_7$	21.3	66.7	65.2	251.07064 (510); 251.07287 (444); 280.07557 (411); 357.09479 (811); 357.09744 (948); 357.10009 (471); 357.10274 (479)

19	Thermopsoside, crotonoyl	7.81	529.135	529.132	5.96	529.135	$C_{26}H_{26}O_{12}$	14.7	75	64.2	243.06405 (523); 243.06623 (573); 529.13754 (697)
20	Taxifolin	7.92	303.051	303.049	6.70	303.051	$C_{15}H_{12}O_7$	72.9	82.1	85.4	125.02128 (205); 125.02284 (478); 125.02441 (392); 125.02598 (153); 125.02755 (120); 150.03253 (164); 285.03616 (128)
21	Kaempferol	7.92	285.040	285.040	2.33	285.040	$C_{15}H_{10}O_6$	4.2	100	62.5	61.98895 (10); 65.00795 (10); 79.96034 (10); 80.96735 (10); 121.02899 (10); 125.10913 (10); 126.68208 (10); 133.02686 (10); 133.06733 (10); 143.05281 (10); 145.03004 (10); 146.07123 (10); 148.0367 (10); 149.09204 (10); 153.05211 (10); 155.11103 (10); 160.08583 (10); 160.58691 (10); 161.09235 (24); 161.09413 (12); 161.09591 (24); 161.09769 (12); 161.10303 (12); 161.10481 (12); 173.0823 (10); 174.03254 (10); 175.03365 (12); 175.04664 (12); 176.981 (10); 179.11562 (10); 180.0766 (10); 183.78642 (10); 195.06835 (10); 195.13499 (10); 197.11989 (10); 199.04355 (12); 199.04553 (12); 201.01435 (12); 201.02231 (12); 202.99286 (10); 205.08153 (12); 205.08957 (24); 217.05171 (10); 223.13057 (10); 226.0601 (10); 239.12666

											(10); 245.47649 (10); 285.03845 (12); 285.04082 (12); 285.04556 (12); 285.05504 (12); 285.09295 (12); 285.11901 (12); 285.12375 (12); 285.13323 (12); 285.16877 (12)
22	Laricitrin	8.02	331.046	331.042	10.71	331.046	$C_{16}H_{12}O_8$	45.9	72.5	69.9	152.01061 (211)
23	Maesopsin	8.02	287.056	287.055	3.53	287.056	$C_{15}H_{12}O_6$	18.1	100	69.8	79.95666 (24); 79.95792 (24); 109.03035 (24); 163.00214 (24); 173.05849 (48) 173.06034 (60); 173.06218 (48); 173.06403 (36); 174.06606 (36); 174.06791 (24); 174.06976 (48); 174.07161 (24); 174.07531 (24); 189.08831 (63); 189.09024 (75); 189.09217 (126); 189.0941 (36); 189.09603 (73); 197.05904 (24); 207.09629 (24); 287.05139 (24); 287.05614 (60); 287.0609 (24); 287.06565 (36)
24	2'-Hydroxy-3,4,5'-trimethoxychalcone	8.24	313.108	313.108	1.43	313.108	$C_{18}H_{18}O_5$	10.8	100	66.9	225.05042 (114); 237.05212 (85); 254.05428 (105); 254.05651 (134); 255.0618 (90); 255.06404 (123); 255.06628 (101); 255.06852 (105); 269.07711 (116); 269.07941 (148); 269.08172 (162);
25	Hyperin	8.45	463.088	463.087	3.68	463.088	$C_{21}H_{20}O_{12}$	11.7	100	67.4	315.00918 (367); 315.01167 (465); 315.01416 (372); 315.01665 (334); 330.0308 (505); 330.03335 (759);

											330.03589 (997); 330.03844 (562); 463.08584 (380)
26	Quercitrin	8.60	447.093	447.090	8.47	447.093	$C_{21}H_{20}O_{11}$	69.5	100	82.1	227.03201 (1941); 227.03413 (2375); 227.03624 (2238); 255.02426 (1448); 255.0265 (2422); 255.02874 (3706); 255.03098 (2387); 255.03322(1844); 284.02574 (1094); 284.02811 (2340); 284.03047 (3839); 284.03284 (3748); 284.0352 (3105); 284.03757 (1348); 285.03886 (1250); 285.04122 (1157); 447.08599 (2132); 447.08896 (2918); 447.09193 (2894); 447.09489 (3145); 447.09786 (1668); 447.10083 (1129)
27	Syringetin-3-O-galactoside	8.73	507.114	507.115	1.74	507.114	$C_{23}H_{24}O_{13}$	82.4	100	93.5	273.03956 (1126); 301.03563 (944); 344.04724 (1205); 344.04984 (1924); 344.05244 (2864); 344.05505 (2570); 344.05765 (1499); 344.06025 (1019); 507.10487 (1332); 507.10803 (1843); 507.11119 (3221); 507.11435 (3755); 507.11751 (3085); 507.12067 (2325); 507.12383 (1766)
28	Isorhamnetin 3-galactoside	8.73	477.104	477.100	7.66	477.104	$C_{22}H_{22}O_{12}$	36.7	86.7	72.2	314.04079 (388); 477.0991 (496); 477.10216 (421); 477.10523 (493); 477.1083 (514)

29	Homoeriodictyol 7-neohesperidoside	8.77	609.182	609.182	0.65	609.183	$C_{28}H_{34}O_{15}$	17.4	100	66.5	285.0739 (586); 285.07627 (550); 285.07864 (398); 609.17103 (524); 609.1745 (479); 609.17796 (591); 609.18142 (474); 609.18489 (595); 609.18835 (395)
30	Procyanidin A1	8.97	575.120	575.121	1.73	575.120	$C_{30}H_{24}O_{12}$	19.6	58.1	60.3	575.11585 (409)
31	Rhoifolin	8.97	577.156	577.155	3.09	577.156	$C_{27}H_{30}O_{14}$	3	100	64.5	315.12112 (1270); 315.12362 (1228);413.11749 (1052); 413.12034 (1237); 413.12319 (1520); 431.12752 (1283); 431.13044 (1736); 431.13335 (1436); 431.13626 (1386); 461.13689 (1552); 461.1399 (3395); 461.14291 (3563); 461.14593 (3594); 461.14894 (1887)
32	Engeletin	9.12	433.114	433.112	4.67	433.114	$C_{21}H_{22}O_{10}$	37.7	75	70.9	253.04454 (701); 253.04677 (1283); 253.049 (2284); 253.05123 (1959); 253.05347 (1552); 253.0557 (606); 433.10535 (712); 433.10827 (1125); 433.11119 (1602); 433.11411 (1536); 433.11703 (1813); 433.11996 (1118); 433.12288 (603)
33	(-)-Aromadendrin	9.27	287.056	287.055	3.53	287.056	$C_{15}H_{12}O_6$	78	71.9	86.2	57.03412 (95); 125.02129 (213); 125.02286 (252); 125.02443 (371); 125.026 (217); 125.02757 (113); 125.02914 (126); 151.00356 (101); 177.05201 (108); 177.05388 (142);

34	Phlorizin	9.29	435.130	435.127	5.92	435.130	$C_{21}H_{24}O_{10}$	44.5	90.9	77	259.05791 (164); 259.06017 (140); 259.06243 (132); 259.06469 (148); 167.03103 (292); 167.03285 (565); 167.03466 (771); 167.03647 (534); 273.07239 (426); 273.07471 (1065); 273.07703 (1093); 273.07935 (671); 273.08167 (339)
35	kaempferol-7-O-hexoside	9.29	447.093	447.087	14.51	447.089	$C_{21}H_{20}O_{11}$	26.5	62.5	65.8	314.03853 (435); 314.04102 (468); 314.0435 (504); 314.04599 (323); 447.08554 (312); 447.08851 (440); 447.09148 (470); 447.09444 (333)
36	Myricetin	9.32	317.030	317.029	4.71	317.030	$C_{15}H_{10}O_8$	76.2	85.7	86.9	107.01181 (218); 109.0274 (176); 109.02887 (337); 109.03033 (209); 137.02027 (303); 137.02192 (410); 137.02356 (603); 137.0252 (259); 137.02684 (253); 151.00019 (374); 151.00192 (968); 151.00364 (506); 151.00537 (290); 151.00709 (208); 178.99561 (197); 178.99749 (263); 178.99936 (213); 179.00124 (255); 317.02246 (180); 317.02495 (208); 317.02745 (331); 317.02995 (438); 317.03245 (270)
37	Juglanin	9.34	417.083	417.079	9.41	417.083	$C_{20}H_{18}O_{10}$	50.9	100	77.9	227.02983 (437); 227.03195 (521); 227.03406 (703); 227.03618 (702); 255.02418 (505); 255.02642 (716); 255.02866 (729); 255.03091 (577); 284.02802 (943); 284.03039 (815); 284.03275 (893); 284.03512 (526); 285.0364 (555); 285.03877 (619)

38	Osajin	9.38	403.155	403.157	4.47	403.155	$C_{25}H_{24}O_5$	13	100	64.8	87.00825 (489); 87.00956 (459); 111.00554 (664); 111.00702 (1264); 111.0085 (1591); 111.00998 (1063); 111.01146 (493)
39	Kaempferol-3-O-alpha-L-rhamnoside	9.58	431.098	431.094	10.84	431.098	$C_{21}H_{20}O_{10}$	69.1	100	85.3	227.03172 (482); 227.03384 (590); 227.03595 (620); 255.02617 (539); 255.02841 (736); 255.03065 (837); 255.03289 (490); 284.02774 (852); 284.03011 (1163); 284.03247 (1276); 284.03484 (532); 284.0372 (474); 285.03375 (747); 285.03612 (1032); 285.03849 (1457); 285.04086 (1073); 285.04323 (816); 285.0456 (504); 431.09257 (476);
40	Pinoquercetin	9.58	315.051	315.048	10.57	315.051	$C_{16}H_{12}O_7$	34.3	56.5	62.5	93.03483 (60); 121.02592 (48); 165.01658 (48); 165.01838 (93); 165.02019 (64); 165.0256 (48); 255.03292 (48); 259.06238 (48); 287.05351 (48)
41	5,7,3',4',5'-Pentahydroxyflavone	9.60	301.035	301.032	10.89	301.035	$C_{15}H_{10}O_7$	75.1	46.7	79.4	149.02011 (129); 149.02182 (320); 149.02354 (173); 149.02525 (301); 149.02696 (153); 149.02868 (77); 149.03039 (67); 151.00181 (73); 301.02596 (90); 301.0284 (185); 301.03083 (275); 301.03327 (442); 301.0357 (253); 301.03814 (387); 301.04057 (91); 301.04301 (77)

42	Tectoridin	9.90	461.109	461.107	3.99	461.109	$C_{22}H_{22}O_{11}$	23.1	100	70.7	241.04874 (529); 241.05092 (605); 269.04043 (425); 269.04273 (671); 269.04503 (698); 269.04734 (490); 269.04964 (412); 298.04341 (667); 298.04583 (796); 298.04825 (747); 298.05068 (636); 461.10057 (531); 461.10359 (943); 461.1066 (879); 461.10961 (799); 461.11263 (703); 461.11564 (577)
43	Cedeodarin	10.36	317.067	317.064	9.08	317.067	$C_{16}H_{14}O_7$	84	90.5	89.9	71.05068 (299); 139.03664 (288); 139.03829 (757); 139.03995 (975); 139.0416 (710); 139.04326 (309); 165.01858 (264); 167.03467 (297); 299.05499 (316); 299.05741 (259)
44	Oroxin A	10.36	431.098	431.097	2.96	431.098	$C_{21}H_{20}O_{10}$	28.5	100	73.1	241.04662 (489); 241.04879 (479); 241.05097 (570); 269.04049 (573); 269.04279 (876); 269.04509 (897); 269.0474 (571); 298.04105 (500); 298.04348 (1043); 298.0459 (1013); 298.04832 (779); 298.05074 (574); 298.05317 (522); 431.0896 (437); 431.09251 (468); 431.09542 (891); 431.09834 (611); 431.10125 (574)
45	Iridin	10.82	521.130	521.128	4.16	521.130	$C_{24}H_{26}O_{13}$	44.4	87.5	72.7	329.02453 (989); 329.02707 (1488); 329.02962 (1698); 329.03216 (1166); 329.03471 (906); 344.04739 (865); 344.04999 (1683); 344.05259 (1394); 344.05519 (1032); 344.0578 (629);

											359.06781 (771); 359.07047 (1308); 359.07313 (2437); 359.07578 (2452); 359.07844 (2252); 359.0811 (1707); 359.08376 (967)
46	Luteolin	10.99	285.040	285.037	11.45	285.040	$C_{15}H_{10}O_6$	67.4	66.7	79.9	65.00566 (10); 83.01176 (10); 93.03341 (10); 113.09993 (10); 121.01967 (12); 121.03048 (12); 132.98958 (10); 133.02357 (24); 133.0268 (24); 133.02842 (24); 133.03004 (115); 133.03166 (24); 133.03328 (12); 134.2207 (10); 135.45586 (10); 139.11582 (10); 149.02344 (24); 149.02515 (12); 149.02858 (12); 150.99999 (12); 151.00516 (12); 153.09892 (10); 166.61387 (10); 171.04637 (10); 175.03357 (12); 175.04471 (12); 183.04895 (10); 189.05135 (10); 197.09419 (10); 198.03307 (10); 199.03753 (10); 201.02023 (10); 227.12464 (10); 241.07266 (12); 241.09445 (12); 243.02884 (10); 250.03091 (10); 257.04241 (10); 284.15058 (10); 285.02886 (24); 285.03596 (12); 285.03833 (66); 285.0407 (89); 285.04307 (36); 285.04544 (36); 285.11178 (12);
47	Tiliroside	11.01	593.130	593.125	8.21	593.130	$C_{30}H_{26}O_{13}$	87.9	100	93.5	284.02735 (2464); 284.02971 (3874); 284.03208 (4916);

											284.03444 (3430); 284.03681 (2266); 285.03572 (2989); 285.03809 (5135); 285.04046 (5504); 285.04283 (3765); 285.0452 (2213); 593.12027 (2845); 593.12369 (4363); 593.12711 (5659); 593.13053 (6304); 593.13395 (5772); 593.13736 (3682); 593.14078 (2477); 593.1442 (1437)
48	Quercetin	11.03	301.035	301.035	2.92	301.035	$C_{15}H_{10}O_7$	87.5	100	93.6	107.01302 (354); 121.02574 (294); 121.02729 (604); 121.02883 (471); 121.03038 (433); 150.99813 (312); 150.99986 (901); 151.00158 (1491); 151.00331 (1593); 151.00503 (1326); 151.00676 (446); 178.99709 (444); 178.99896 (287); 301.03039 (470); 301.03282 (552); 301.03526 (466)
49	Wogonoside	11.03	459.093	459.092	3.68	459.093	$C_{22}H_{20}O_{11}$	10.7	100	68.4	227.03161 (181); 255.02604 (227); 255.02828 (194); 284.0276 (280); 284.02996 (374); 284.03233 (342); 284.03469 (278); 284.03706 (254)
50	Isorhamnetin	11.12	315.051	315.048	11.20	315.051	$C_{16}H_{12}O_7$	16	100	69.3	271.0585 (134); 271.06312 (160); 297.0319 (129); 297.03674 (272); 297.03916 (494); 297.04158 (351); 297.04399 (215); 297.04641 (134); 300.02629 (190); 300.02872 (155); 315.0491 (134);

51	Hesperetin	11.61	301.072	301.071	3.87	301.072	$C_{16}H_{14}O_6$	4.2	100	61.5	139.03628 (471); 139.03794 (1026); 139.03959 (1116); 139.04124 (854); 139.0429 (526); 139.04455 (278); 273.07402 (356); 273.07634 (549); 273.07866 (341)
52	Chamaechromone	11.69	541.114	541.110	6.70	541.114	$C_{30}H_{22}O_{10}$	18.8	61.5	61	111.00675 (1172); 111.00823 (1339); 111.00971 (1209); 285.03786 (1019); 285.04023 (1429); 285.0426 (1111); 313.03141 (1701); 313.03389 (2228); 313.03637 (1886); 313.03886 (1238); 328.05461 (1799); 328.05715 (2211); 328.05969 (1953); 328.06224 (1337); 343.07633 (1462); 343.07893 (2969); 343.08153 (3183); 343.08413 (3031); 343.08673 (1811); 343.08933 (1069); 505.1307 (1317); 505.13385 (1336)
53	Glyasperin F	12.13	353.103	353.106	7.18	353.103	$C_{20}H_{18}O_6$	29.5	50	66.3	229.15764 (138); 229.16189 (205); 273.14187 (130); 273.14419 (253); 273.14651 (289); 273.14883 (420); 273.15115 (367); 273.15347 (318); 273.15579 (196); 353.09938 (209); 353.10202 (236); 353.10466 (419); 353.10729 (564); 353.10993 (351); 353.11257 (377); 353.22597 (142); 353.2286 (143); 353.23124 (152);

											353.23388 (330); 353.23652 (135); 353.23915 (143)
54	Phloretin	12.31	273.077	273.074	12.26	273.077	$C_{15}H_{14}O_5$	84.8	42.9	81.8	81.03183 (12); 81.03309 (12); 81.03435 (36); 81.03562 (24); 119.04729 (24); 119.04882 (48); 119.05035 (24); 119.05188 (24); 123.02723 (12); 123.03812 (12); 123.04279 (36); 123.04435 (76); 123.04746 (12); 123.04902 (24); 123.0568 (12); 151.00017 (24); 151.00189 (12); 151.00706 (12); 167.02731 (12); 167.02913 (12); 167.03094 (12); 167.03275 (60); 167.03457 (74); 167.03638 (103); 167.04001 (12); 167.04545 (24); 167.04908 (12); 187.06944 (12); 187.07328 (12); 187.07904 (12); 189.05158 (12); 189.05351 (24); 189.05544 (24); 255.05995 (12); 255.0734 (12); 273.07688 (24); 273.0792 (12)
55	Theaflavin	12.78	563.120	563.119	1.43	563.120	$C_{29}H_{24}O_{12}$	37.3	75	70.4	255.02824 (13535); 255.03048 (14643); 255.03273 (10748); 284.02756 (18743); 284.02992 (43971); 284.03229 (68692); 284.03465 (57504); 284.03702 (31853); 284.03938 (14416); 285.03593 (11427); 285.0383 (22149); 285.04067 (25895); 285.04304 (19816); 285.04541

											(11694); 563.11077 (19728); 563.1141 (33130); 563.11743 (47989); 563.12076 (60700); 563.12409 (46436); 563.12742 (32745); 563.13075 (15972)
56	4',5,7-Trihydroxy 3,6,8-trimethoxyflavone	13.32	359.077	359.073	10.71	359.077	$C_{18}H_{16}O_8$	16.6	66.7	63.5	302.03442 (392); 302.03686 (829); 302.0393 (1510); 302.04174 (2345); 302.04417 (2333); 302.04661 (1702); 302.04905 (956); 302.05149 (510); 344.04951 (264); 344.05211 (423); 344.05471 (441); 359.07528 (253)
57	Procyanidin B1	14.07	577.135	577.130	9.28	577.135	$C_{30}H_{26}O_{12}$	50.4	42.3	62.7	269.04175 (615); 269.04405 (693); 269.04635 (641); 298.04232 (1406); 298.04474 (2393); 298.04716 (2745); 298.04959 (2047); 298.05201 (1385); 298.05443 (653); 299.05098 (887); 299.05341 (1336); 299.05584 (1285); 299.05826 (803); 577.12103 (748); 577.1244 (1112); 577.12777 (1862); 577.13114 (2108); 577.13451 (2666); 577.13788 (2383); 577.14126 (1907); 577.14463 (1065)

58	Tricin methyl ether	15.50	343.082	343.081	5.33	343.082	$C_{18}H_{16}O_7$	55.7	44.4	73.5	285.03569 (36); 313.02914 (75); 313.03162 (122); 313.03411 (150); 313.03659 (89); 313.03907 (83); 328.05484 (48); 343.07917 (36)
59	3-Hydroxy-6,3',4'-trimethoxyflavone	18.16	327.087	327.084	9.52	327.087	$C_{18}H_{16}O_6$	80.9	57.1	84.2	269.03727 (65); 269.03957 (121); 269.04188 (133); 269.04418 (146); 269.04648 (134); 269.04878 (118); 269.05108 (60); 297.03308 (104); 297.0355 (129); 297.03792 (520); 297.04034 (458); 297.04275 (244); 297.04517 (216); 297.04759 (106); 297.05001 (60); 312.05849 (60); 312.06097 (122); 312.06345 (150); 312.06593 (168); 312.06841 (96)
60	6-Hydroxy-3',4',5,7-tetramethoxyflavone	18.16	357.098	357.096	4.70	357.098	$C_{19}H_{18}O_7$	56.6	54.5	74.8	284.02632 (109); 284.02868 (213); 284.03105 (228); 284.03341 (134); 312.01837 (120); 312.02085 (225); 312.02333 (273); 312.02581 (248); 312.02829 (237); 312.03076 (130); 312.03324 (118); 327.04289 (163); 327.04542 (264); 327.04796 (278); 327.0505 (285); 327.05304 (388); 327.05558 (149); 327.05811 (127); 342.07021 (112); 342.07281 (243); 342.07541 (128)

Table S2. Identification of flavonoids from purified extracts of PN in the CK1 group by UHPLC-MS/MS in negative ion mode ([M-H]⁻).

No.	Title	RT (min)	theoretical m/z	Precursor m/z	PPM	Reference m/z	Formula	DP	FP (%)	TS	MS/MS Fragments
1	Dichamanetin	4.37	467.150	467.151	2.97	467.150	$C_{29}H_{24}O_6$	15.7	66.7	61.9	165.05439 (171); 467.15043 (186); 467.15347 (224); 467.1565 (355); 467.15953 (233); 467.16257 (190)
2	Phloretin 2-O-xyloglucoside	4.43	567.172	567.169	5.35	567.172	$C_{26}H_{32}O_{14}$	24	100	69.8	567.16129 (462); 567.16464 (534); 567.16798 (885); 567.17132 (888); 567.17466 (1055); 567.178 (1023); 567.18135 (669)
3	Padmatin	4.54	317.067	317.066	1.20	317.067	$C_{16}H_{14}O_7$	18.3	44.4	63.1	59.01252 (36); 141.03576 (36); 176.03387 (99); 176.03574 (63); 176.0376 (73); 317.06725 (48)
4	Isobavachalcone	4.61	323.129	323.132	10.26	323.129	$C_{20}H_{20}O_4$	20.3	100	65	75.00884 (48); 145.08426 (89); 145.08596 (200); 145.08765 (100); 145.09103 (71); 145.09272 (48); 177.03908 (74); 323.13595 (48)
5	Andrographidin B	4.71	491.120	491.118	2.86	491.120	$C_{23}H_{24}O_{12}$	36.4	100	73.7	123.04419 (1112); 125.02275 (1685); 125.02432 (1859); 125.02589 (992); 167.03079 (1849); 167.0326 (4761); 167.03442 (6668); 167.03623 (4601); 167.03804 (2726); 167.03986 (1326); 491.13248 (973); 491.13559 (2191); 491.1387 (2940); 491.14181 (3501); 491.14492 (2544); 491.14803 (1602); 491.15425 (1078)

6	ROTENONIC ACID, METHYL ETHER	4.76	409.166	409.167	2.29	409.166	$C_{24}H_{26}O_6$	10.2	60	60.7	137.05965 (5210)
7	7-O-Benzylluteolin	5.03	375.087	375.092	13.03	375.087	$C_{22}H_{16}O_6$	20.8	66.7	62	154.02754 (719); 178.99541 (872); 178.99728 (2119); 178.99916 (2417); 179.00104 (2584); 179.00292 (1151); 197.00562 (1101); 197.00759 (1954); 197.00956 (2281); 197.01153 (2394); 197.0135 (1066); 212.03037 (1046); 212.03241 (2175); 212.03445 (1964); 212.0365 (1672); 375.09103 (1063); 375.09374 (1689); 375.09646 (1708); 375.09918 (1374); 375.1019 (968)
8	(-)-Epicatechin	5.14	289.072	289.072	1.16	289.072	$C_{15}H_{14}O_6$	74.2	84.4	86.1	109.02864 (70); 109.03011 (106); 121.02732 (70); 123.04103 (97); 123.04258 (136); 123.04414 (292); 123.04725 (167); 125.0227 (94); 151.03961 (73)
9	Isotriuvaretin	5.81	589.223	589.222	1.66	589.223	$C_{37}H_{34}O_7$	19.9	73.9	61.5	201.10999 (480); 201.11198 (775); 201.11397 (755); 201.11596 (529); 271.0955 (703); 271.09781 (803)
10	Hovetrichoside C	6.07	449.109	449.107	5.21	449.109	$C_{21}H_{22}O_{11}$	53.2	100	81.9	125.02278 (364); 259.05794 (318); 259.0602 (505); 259.06246 (620); 259.06472 (464); 259.06698 (384); 269.04524 (482); 269.04754 (419); 269.04985 (355); 287.056 (325); 287.05837 (329); 449.10871 (412); 449.11169 (312); 449.11466 (341)

11	3'-Hydroxy-.alpha.-naphthoflavone	6.07	287.071	287.069	8.25	287.071	$C_{19}H_{12}O_3$	30.4	75	67.9	144.04637 (142); 173.03607 (272); 173.03792 (147); 183.05275 (218); 183.05465 (427); 183.05655 (449); 183.05845 (250); 183.06035 (163); 215.0406 (150); 215.04266 (434); 215.04472 (572); 215.04678 (662); 215.04883 (418); 215.05089 (222); 243.07705 (172); 287.06059 (250); 287.06297 (334); 287.06535 (765); 287.06772 (839); 287.0701 (577); 287.07248 (242); 287.07486 (197)
12	Cedrin	6.15	333.062	333.062	2.12	333.062	$C_{16}H_{14}O_8$	20	80	60.9	163.00001 (336); 163.0018 (728); 163.00359 (766); 163.00538 (360); 163.00717 (305); 315.04625 (380); 315.04874 (786); 315.05123 (800); 315.05372 (640); 315.05622 (466)
13	Epimedin I	6.47	879.293	879.296	3.94	879.293	$C_{41}H_{52}O_{21}$	1.2	100	60.8	207.04558 (1044); 207.0476 (2732); 207.04962 (4260); 207.05164 (3653); 207.05365 (2457); 207.05567 (1225); 207.05769 (571); 353.0781 (631); 353.08074 (1378); 353.08337 (2621); 353.08601 (3332); 353.08865 (3124); 353.09128 (2149); 353.09392 (1369); 353.09656 (751); 353.09919 (542)
14	Bonaniol A	6.79	423.181	423.185	9.18	423.181	$C_{25}H_{28}O_6$	28.1	60	60.9	117.05425 (884); 117.05577 (845); 117.05729 (566); 199.09681 (621); 199.09879 (628); 423.18062 (710); 423.18351 (993); 423.1864 (1159);

											423.18928 (936); 423.19217 (975); 423.19506 (565)
15	Anhydroicaritin 3-(2-rhamnosylrhamnoside)	6.84	659.235	659.236	1.62	659.235	$C_{33}H_{40}O_{14}$	45.5	50	70.3	167.03079 (1071); 167.0326 (2897); 167.03441 (3996); 167.03623 (3075); 167.03804 (1983); 167.03985 (919); 659.25289 (1191); 659.2565 (1287); 659.2601 (1513); 659.2637 (1957); 659.26731 (2138); 659.27091 (2695); 659.27451 (2371); 659.27812 (1683); 659.28172 (1141)
16	Procyanidin A2	7.12	575.120	575.124	7.30	575.120	$C_{30}H_{24}O_{12}$	25.7	84.4	68.1	287.04876 (898); 287.05113 (1241); 287.05351 (1460); 287.05589 (1562); 287.05826 (1218); 287.06064 (998)
17	Marein	7.14	449.109	449.110	2.36	449.109	$C_{21}H_{22}O_{11}$	15.5	100	68.9	151.00182 (413); 151.00354 (332); 269.04073 (363); 269.04303 (609); 269.04533 (660); 269.04763 (555); 269.04994 (344); 449.10589 (354); 449.10886 (399); 449.11184 (351); 449.11481 (346)
18	Isobavachin	7.50	323.129	323.124	14.19	323.129	$C_{20}H_{20}O_4$	12.5	100	65.4	161.07441 (48); 323.14543 (48)
19	Kuwanon C	7.53	421.166	421.167	2.93	421.167	$C_{25}H_{26}O_6$	30	100	70.9	183.10198 (662); 243.12062 (998); 243.12281 (1324); 243.125 (1277); 421.16227 (1064); 421.16515 (1415); 421.16803 (2229); 421.17091 (2516); 421.17379 (2215); 421.17667 (931); 421.17955 (810); 421.18243 (786)

20	Miquelianin	7.59	477.067	477.067	0.56	477.068	$C_{21}H_{18}O_{13}$	15	75	66	300.02375 (375); 300.02618 (515); 300.02861 (357); 301.03333 (367)
21	Hirsutrin	7.76	463.088	463.087	2.60	463.088	$C_{21}H_{20}O_{12}$	54.1	100	84.1	271.0239 (538); 271.02621 (610); 300.02151 (670); 300.02395 (1010); 300.02638 (1321); 300.02881 (1208); 300.03124 (692); 301.03109 (524); 301.03352 (582); 301.03596 (583); 463.08018 (437); 463.08622 (645); 463.08924 (636); 463.09226 (438)
22	Laricitrin 3-galactoside	7.80	493.099	493.097	2.78	493.099	$C_{22}H_{22}O_{13}$	10	100	63.7	315.01658 (417)
23	dihydroquercetin	7.86	303.051	303.049	6.04	303.051	$C_{15}H_{12}O_{7}$	62.1	53.5	74.1	57.03501 (48); 123.04408 (36); 125.0195 (36); 125.02263 (94); 125.0242 (83); 125.02577 (70); 125.02891 (48); 125.03048 (36); 150.02888 (36); 150.03232 (36); 151.00332 (48); 175.03718 (48); 175.03904 (60); 175.04089 (36); 285.04066 (36); 285.04303 (36); 285.0454 (48)
24	Artocaprin	7.94	435.181	435.185	9.39	435.183	$C_{26}H_{28}O_{6}$	47.4	50	69.4	155.10398 (1782); 155.10573 (3160); 155.10748 (4416); 155.10922 (3930); 155.11097 (1987); 155.11272 (984); 199.09501 (1078); 199.09699 (1667); 199.09897 (1386); 199.10095 (793); 435.18148 (1913); 435.18441 (3888); 435.18734 (4243);

											435.19026 (3641); 435.19319 (2473); 435.19612 (1505)
25	Quercetin-3-O-xyloside	8.42	433.078	433.078	0.32	433.078	$C_{20}H_{18}O_{11}$	94.8	90.6	94.4	255.02823 (5076); 255.03047 (4077); 271.02118 (7314); 271.02349 (10714); 271.0258 (8964); 271.02811 (5991); 300.02107 (5676); 300.0235 (13883); 300.02593 (23569); 300.02836 (26142); 300.03079 (18515); 300.03322 (10490); 300.03565 (4946); 301.03064 (9467); 301.03307 (15109); 301.03551 (17697); 301.03794 (12668); 301.04038 (6665); 433.07307 (4930); 433.07599 (6136); 433.07891 (7120)
26	Astragalin	8.50	447.093	447.090	7.36	447.093	$C_{21}H_{20}O_{11}$	76.3	100	85.4	227.03171 (4675); 227.03383 (8321); 227.03594 (7788); 227.03806 (4909); 255.02619 (5610); 255.02843 (9692); 255.03067 (10022); 255.03291 (6868); 255.03515 (3952); 284.02779 (5531); 284.03015 (12582); 284.03252 (15706); 284.03488 (13498); 284.03725 (7624); 284.03961 (3614); 285.03854 (4564); 285.04091 (4743); 447.08564 (4247); 447.08861 (6902); 447.09158 (11542); 447.09454 (11117);

											447.09751 (8590); 447.10048 (5034); 447.10345 (2929)
27	Syringetin-3-O-galactoside	8.61	507.114	507.113	3.19	507.114	$C_{23}H_{24}O_{13}$	84.6	100	94.2	273.03739 (1403); 273.03971 (1768); 344.04749 (1785); 344.0501 (2811); 344.0527 (4474); 344.0553 (4095); 344.0579 (2603); 344.06051 (1547); 507.10854 (2135); 507.1117 (4925); 507.11486 (5754); 507.11802 (4785); 507.12118 (4680); 507.12434 (3154); 507.1275 (1583)
28	Isorhamnetin 3-galactoside	8.65	477.104	477.100	7.24	477.104	$C_{22}H_{22}O_{12}$	68.9	93.3	84.6	243.02884 (950); 243.03103 (882); 271.02585 (721); 314.03834 (709); 314.04083 (1339); 314.04331 (1513); 314.0458 (1215); 314.04829 (1011); 314.05077 (743); 477.09623 (892); 477.0993 (1381); 477.10236 (1810); 477.10543 (2346); 477.10849 (2586); 477.11156 (1324); 477.11462 (850)
29	Hesperetin-7-O-neohesperidoside	8.65	609.182	609.183	0.17	609.183	$C_{28}H_{34}O_{15}$	14.1	100	65.8	285.07657 (416); 285.07894 (417); 609.17893 (396); 609.18586 (443); 609.18932 (426)
30	Sophoricoside	8.70	431.098	431.099	2.38	431.098	$C_{21}H_{20}O_{10}$	27.9	100	71.5	268.0355 (370); 268.03779 (303); 268.04009 (395); 431.09547 (290); 431.09838 (341); 431.10129 (249); 431.10421 (275)
31	Isorhoifolin	8.83	577.156	577.154	3.61	577.156	$C_{27}H_{30}O_{14}$	1.3	100	63.8	413.12284 (738); 431.13008 (714); 431.133 (912); 431.13591 (913);

										461.13653 (681); 461.13955 (1051); 461.14256 (1953); 461.14557 (1613); 461.14859 (1325); 461.1516 (914)
32	4',5,7-Trihydroxy 3,3',6,8-tetramethoxyflavone	8.89	389.088	389.084	10.82	389.088	$C_{19}H_{18}O_9$	9.4	100	63.7	136.01327 (387); 136.01491 (519); 136.01654 (412); 136.01818 (316); 151.03784 (321); 151.03956 (341)
33	Hemiphloin	9.00	433.114	433.112	3.75	433.114	$C_{21}H_{22}O_{10}$	36	75	68.9	253.04443 (575); 253.04666 (1143); 253.04889 (2094); 253.05112 (1538); 253.05336 (1117); 253.05559 (549); 433.10535 (685); 433.10827 (1284); 433.11119 (1630); 433.11411 (1964); 433.11703 (1725); 433.11995 (880); 433.12287 (423)
34	(-)-Aromadendrin	9.15	287.056	287.055	5.62	287.056	$C_{15}H_{12}O_6$	62.8	53.1	75.8	65.00443 (48); 125.01956 (48); 125.02113 (60); 125.02269 (141); 125.02426 (68); 125.02583 (60); 125.0274 (48); 177.05745 (60); 259.05777 (78); 259.06228 (48)
35	p-Phlorizin	9.18	435.130	435.130	0.40	435.130	$C_{21}H_{24}O_{10}$	47.8	100	80.3	123.04403 (413); 125.02102 (439); 125.02259 (473); 167.03057 (470); 167.03238 (856); 167.0342 (1014); 167.03601 (732); 273.07177 (844); 273.07409 (1584); 273.0764 (1749); 273.07872 (1221); 273.08104 (599); 435.0873 (380); 435.09316 (652)
36	Kaempferol-3-O-alpha-L-arabinoside	9.24	417.083	417.084	2.82	417.083	$C_{20}H_{18}O_{10}$	88.3	100	94	227.03343 (9231); 227.03554 (14825); 227.03766 (15852); 227.03977 (7825); 255.02798

											(10268); 255.03022 (19434); 255.03246 (17970); 255.0347 (14165); 255.03694 (7424); 284.02965 (9998); 284.03202 (21752); 284.03438 (29293); 284.03675 (24192); 284.03911 (12595); 284.04148 (5677); 285.03804 (7324); 285.0404 (12840); 285.04277 (14001); 285.04514 (10885); 285.04751 (6006); 417.08247 (6549); 417.08534 (8342); 417.0882 (7101)
37	Kaempferol	9.24	285.040	285.040	3.38	285.040	$C_{15}H_{10}O_6$	5.8	100	62.9	59.0178 (10); 79.02175 (10); 101.03658 (10); 119.04844 (12); 119.04998 (36); 119.05151 (24); 119.05304 (12); 125.02566 (10); 141.09217 (10); 143.04914 (10); 154.03773 (10); 155.04779 (10); 166.1032 (10); 171.04244 (12); 171.04611 (12); 171.04978 (12); 171.05161 (12); 172.1196 (10); 179.15284 (10); 186.03012 (10); 193.06423 (10); 197.05846 (10); 211.04012 (12); 211.06867 (12); 219.10291 (10); 229.0487 (10); 239.03297 (10); 259.0687 (10); 267.14679 (10); 285.02619 (12); 285.0404 (12); 285.04988 (12); 285.1731 (10)

38	Kaempferol-3-O-alpha-L-rhamnoside	9.44	431.098	431.096	4.58	431.098	$C_{21}H_{20}O_{10}$	58.4	90.9	83.4	227.03569 (612); 227.0378 (518); 255.02814 (480); 255.03038 (848); 255.03262 (650); 284.02747 (482); 284.02983 (745); 284.0322 (1166); 284.03456 (1175); 284.03693 (597); 284.03929 (521); 285.03585 (489); 285.03822 (1072); 285.04059 (1122); 285.04296 (936); 285.04532 (546)
39	petunidin-3-O-arabinoside	9.53	447.093	447.093	1.54	447.093	$C_{21}H_{20}O_{11}$	63.8	64.7	76.9	243.02669 (1071); 243.02887 (1619); 243.03106 (1722); 271.02127 (1398); 271.02358 (1512); 271.02589 (1784); 271.0282 (1121); 285.03841 (1004); 285.04078 (1271); 314.0359 (991); 314.03838 (1714); 314.04087 (2840); 314.04336 (3149); 314.04584 (2546); 314.04833 (2124); 447.08841 (1976); 447.09138 (2221); 447.09435 (2252); 447.09732 (1793); 447.10028 (1360); 447.10325 (1032)
40	Liquiritin	9.55	417.119	417.119	0.98	417.119	$C_{21}H_{22}O_{9}$	34.6	83.3	74.4	227.03343 (9231); 227.03554 (14825); 227.03766 (15852); 227.03977 (7825); 255.02798 (10268); 255.03022 (19434); 255.03246 (17970); 255.0347 (14165); 255.03694 (7424); 284.02965 (9998); 284.03202 (21752); 284.03438 (29293);

											284.03675 (24192); 284.03911 (12595); 284.04148 (5677); 285.03804 (7324); 285.0404 (12840); 285.04277 (14001); 285.04514 (10885); 285.04751 (6006); 417.08247 (6549); 417.08534 (8342); 417.0882 (7101)
41	Coatline B	9.63	451.125	451.128	7.78	451.125	$C_{21}H_{24}O_{11}$	30.1	50	63.7	96.95584 (226); 96.95722 (1008); 96.9586 (1625); 96.95998 (1718); 96.96136 (1263); 96.96274 (454); 96.96413 (411); 451.12141 (229); 451.12439 (309); 451.12737 (510)
42	Tectoridin	9.76	461.109	461.105	8.33	461.109	$C_{22}H_{22}O_{11}$	34.3	100	72.9	241.04667 (2049); 241.04885 (2175); 241.05102 (1487); 241.0532 (1471); 269.04057 (1869); 269.04288 (2436); 269.04518 (2841); 269.04748 (1816); 269.04978 (1398); 298.04117 (1528); 298.04359 (2336); 298.04602 (3560); 298.04844 (4290); 298.05086 (2052); 298.05328 (1225); 461.09799 (1166); 461.10101 (1500); 461.10402 (3408); 461.10703 (3720); 461.11005 (3881); 461.11306 (2648); 461.11607 (1681); 461.11909 (1552)
43	Maritimein	9.92	447.093	447.092	3.55	447.093	$C_{21}H_{20}O_{11}$	15.4	100	69.1	285.03788 (506); 314.03779 (506); 314.04028 (883); 314.04277 (1005); 314.04525 (922); 314.04774 (680);

											315.04575 (478); 315.04824 (779); 315.05073 (928); 315.05322 (634); 447.21814 (512); 447.22111 (677); 447.22407 (651); 447.22704 (657)
44	Apigenin 7-sulfate	10.16	349.002	349.002	2.48	349.002	$C_{15}H_{10}O_8S$	14.2	58.3	60.7	269.04262 (155); 269.04492 (139); 269.04722 (153); 349.19653 (151); 349.19916 (203); 349.20178 (286); 349.2044 (315); 349.20702 (191)
45	Cedeodarin	10.22	317.067	317.065	4.03	317.067	$C_{16}H_{14}O_7$	77	88.1	88.1	139.03628 (213); 139.03794 (317); 139.03959 (492); 139.04125 (269); 139.0429 (222); 139.04456 (136); 139.04621 (162); 165.01999 (140); 167.03065 (140); 167.03428 (141)
46	Cedrusin	10.33	345.134	345.132	6.27	345.134	$C_{19}H_{22}O_6$	2.9	100	61.2	121.02868 (236); 134.03679 (203); 134.03841 (171); 149.06257 (168); 167.06854 (274); 167.07036 (352); 167.07217 (228)
47	2'-Hydroxy-3,4,5'-trimethoxychalcone	10.35	313.108	313.105	10.38	313.108	$C_{18}H_{18}O_5$	6.4	100	64.4	109.03141 (24); 121.02714 (24); 239.06982 (24); 239.07199 (24); 239.07415 (24); 253.08423 (60); 253.08869 (36); 253.09316 (24); 254.0943 (24); 269.08378 (24); 269.17356 (24); 313.10358 (24); 313.20043 (24)
48	3'-Hydroxy-5,7,4',5'-tetramethoxyflavone	10.53	357.098	357.096	5.82	357.098	$C_{19}H_{18}O_7$	2.7	100	61.3	133.02834 (60); 133.02996 (78); 133.03158 (74); 135.04289 (87); 135.04452 (118); 135.04615 (80); 135.04778 (60); 179.03474 (103)
49	Demethoxycentaureidin 7-O-rutinoside	10.64	637.177	637.174	5.99	637.177	$C_{29}H_{34}O_{16}$	16.3	80	64.9	313.03438 (694); 328.0526 (610); 328.05514 (759); 328.05768 (1016);

											328.06022 (863); 328.06276 (641); 343.0743 (621); 343.0769 (775); 343.0795 (1450); 343.0821 (1888); 343.08469 (1689); 343.08729 (995); 637.17322 (651); 637.17677 (791); 637.18031 (1004); 637.18385 (788)
50	Oroxin A	10.66	431.098	431.097	4.12	431.098	$C_{21}H_{20}O_{10}$	28.1	100	72.8	241.04627 (1103); 241.04845 (2374); 241.05063 (2491); 241.05281 (1672); 241.05498 (869); 269.04013 (1437); 269.04243 (2518); 269.04473 (2915); 269.04704 (2362); 269.04934 (1302); 269.05164 (923); 298.0431 (2019); 298.04552 (3601); 298.04795 (4043); 298.05037 (3136); 298.05279 (1541); 298.05522 (1084); 299.05177 (1409); 299.0542 (1961); 299.05662 (1761); 299.05905 (1433); 431.09209 (1094); 431.095 (1446); 431.09792 (1460); 431.10083 (1199); 431.10374 (952)
51	Tiliroside	10.87	593.130	593.128	3.49	593.130	$C_{30}H_{26}O_{13}$	93.2	100	97.1	284.02966 (4778); 284.03203 (7496); 284.03439 (8709); 284.03676 (5743); 284.03912 (3661); 285.03568 (3242); 285.03804 (5941); 285.04041 (10230); 285.04278 (9290); 285.04515 (6038); 285.04752 (3344); 593.1239 (4667); 593.12732

											(8858); 593.13073 (14174); 593.13415 (16963); 593.13757 (12734); 593.14099 (8679); 593.14441 (5137); 593.14782 (2769); 593.15124 (2516)
52	Tamarixetin	10.98	315.051	315.050	3.90	315.051	$C_{16}H_{12}O_7$	19.6	100	70.2	271.05787 (85); 271.06249 (89); 297.03366 (114); 297.03608 (184); 297.0385 (161); 297.04092 (173); 297.04333 (123); 297.04575 (97); 300.02319 (87); 300.02562 (171); 300.02805 (144); 300.03048 (125); 300.03291 (85)
53	Dihydrorobinetin	11.26	303.051	303.049	8.35	303.051	$C_{15}H_{12}O_7$	12.2	66.7	63.1	77.04089 (24); 79.01675 (60); 79.018 (36); 79.01925 (48); 79.02049 (36); 79.02174 (24); 117.07085 (24); 121.02561 (36); 121.02715 (36); 121.02869 (36); 121.03024 (24); 121.03332 (24); 137.0264 (24); 165.01621 (60); 165.01801 (36); 165.01981 (72); 165.02162 (108); 165.02342 (48); 165.02702 (36); 165.03063 (24); 193.01743 (24); 206.02072 (24); 231.07287 (24); 259.06417 (48); 259.06869 (24); 259.14549 (24); 303.04478 (24); 303.04967 (36)
54	Genistein	11.43	269.046	269.044	6.50	269.046	$C_{15}H_{10}O_5$	58.3	42.9	71.4	119.05154 (10); 179.14725 (10); 225.09028 (12); 225.1008 (12); 227.04195 (10); 227.13076 (10);

											269.04246 (12); 269.05167 (12); 269.05627 (12)
55	Hesperetin	11.46	301.072	301.070	4.86	301.072	$C_{16}H_{14}O_6$	5.1	100	61.2	71.04911 (191); 71.05029 (154); 139.03602 (255); 139.03768 (404); 139.03933 (619); 139.04098 (537); 139.04264 (301); 139.04429 (142); 273.07138 (142); 273.0737 (272); 273.07602 (320)
56	5-Glc tricin	11.89	491.120	491.117	4.49	491.121	$C_{23}H_{24}O_{12}$	81.2	89.3	80	269.04248 (20381); 269.04478 (25970); 269.04708 (20365); 285.03576 (23137); 285.03813 (49787); 285.0405 (50906); 285.04287 (35778); 313.03173 (34644); 313.03422 (47524); 313.0367 (41503); 313.03918 (23237); 314.03808 (23490); 314.04056 (42928); 314.04305 (46641); 314.04554 (37131); 314.04802 (22550); 329.0597 (28969); 329.06225 (66449); 329.06479 (125871); 329.06734 (131200); 329.06988 (76999); 329.07243 (45260); 491.11308 (35744); 491.11619 (71311); 491.1193 (67475); 491.12241 (58038); 491.12552 (34300)
57	Myricetin	12.29	317.030	317.028	7.24	317.030	$C_{15}H_{10}O_8$	2.9	100	61.2	163.00361 (48); 189.04937 (48); 189.05323 (60); 189.05516 (48); 192.00131 (48); 192.00325 (134); 192.0052 (82); 192.00714 (130);

											193.00792 (60); 193.00987 (60); 193.01181 (158); 193.01376 (48); 193.01571 (60); 299.04964 (129); 299.05206 (155); 299.05449 (231); 299.05692 (145); 299.05934 (60); 299.06177 (60)
58	Hispiduloside	12.47	461.109	461.110	1.65	461.109	$C_{22}H_{22}O_{11}$	26.9	55.6	63.4	285.03778 (637); 285.04015 (773); 313.02886 (375); 313.03134 (704); 313.03383 (895); 313.03631 (590); 313.03879 (460); 314.04017 (395); 314.04515 (434); 328.0571 (406); 328.05964 (484); 329.05929 (450); 329.06184 (610); 329.06438 (999); 329.06693 (943); 329.06947 (615); 329.07202 (376); 461.10277 (516); 461.10578 (862); 461.10879 (934); 461.11181 (639); 461.11482 (406); 461.11783 (464)
59	Daidzein 4'-sulfate	12.53	333.007	333.005	6.46	333.008	$C_{15}H_{10}O_{7}S$	8.7	100	63.2	253.0462 (219); 253.04844 (428); 253.05067 (296); 253.0529 (262); 287.1599 (192); 287.16228 (317); 287.16465 (201); 287.16703 (226); 333.16033 (213); 333.16289 (439); 333.16545 (979); 333.16801 (1771); 333.17057 (1687); 333.17314 (1370); 333.1757 (959); 333.17826 (403); 333.18082 (315); 333.18338 (189)
60	Iridin	12.58	521.130	521.127	5.12	521.130	$C_{24}H_{26}O_{13}$	67	100	83.6	301.03254 (1577); 301.03498 (1406); 329.02369 (1091);

											329.02624 (1719); 329.02878 (1953); 329.03133 (1886); 344.04913 (1523); 344.05173 (2058); 344.05433 (1944); 344.05694 (1233); 359.06692 (1181); 359.06958 (2349); 359.07224 (4712); 359.0749 (6628); 359.07756 (6712); 359.08022 (4498); 359.08287 (2283); 359.08553 (1281); 521.126 (1136); 521.1292 (1081)
61	Theaflavin	12.64	563.120	563.115	8.35	563.120	$C_{29}H_{24}O_{12}$	35.3	50	64.2	255.02813 (12902); 255.03037 (13342); 255.03261 (9384); 284.02746 (17672); 284.02982 (39033); 284.03219 (61933); 284.03455 (51271); 284.03692 (25802); 284.03928 (12775); 285.03584 (9792); 285.0382 (17603); 285.04057 (22477); 285.04294 (14952); 285.04531 (8846); 563.11085 (15255); 563.11418 (28134); 563.11751 (47323); 563.12084 (48860); 563.12417 (43610); 563.1275 (29303); 563.13083 (13711)
62	3',4',6-Trihydroxy-3,5,7-trimethoxyflavone	13.16	359.077	359.074	8.76	359.077	$C_{18}H_{16}O_8$	17.9	100	69.4	301.03305 (442); 302.03457 (694); 302.03701 (1483); 302.03945 (2924); 302.04189 (3681); 302.04433 (2590); 302.04677 (2012); 302.0492 (1155); 302.05164

										(616); 302.05408 (410); 344.04971 (386); 344.05232 (748); 344.05492 (408); 344.05752 (383)
63	Linarin	13.24	591.172	591.172	0.23	591.172	$C_{28}H_{32}O_{14}$	8.9	100	65.8	329.06228 (384); 329.06482 (709); 329.06737 (587); 329.06991 (300); 591.17088 (331); 591.17429 (332); 591.24254 (359); 591.24595 (372); 591.24936 (407)
64	Isoprunetin	14.65	283.061	283.059	7.42	283.061	$C_{16}H_{12}O_5$	56.4	100	82.2	117.02806 (10); 153.05315 (10); 157.01414 (10); 171.04385 (10); 183.04823 (12); 183.05203 (12); 192.05701 (10); 195.09104 (10); 196.41041 (10); 197.02449 (10); 211.03961 (10); 212.04583 (12); 212.04991 (12); 240.02926 (12); 240.03795 (24); 240.04013 (12); 240.04448 (12); 247.13165 (10); 268.02072 (12); 268.03221 (12); 268.0368 (12); 268.0391 (60); 268.0414 (12); 283.05542 (12); 283.06014 (12); 283.06722 (12); 283.14278 (10); 284.19216 (10)
65	Pectolinarigenin	15.03	313.072	313.067	14.90	313.072	$C_{17}H_{14}O_6$	23.9	66.7	63.8	241.0458 (226); 241.04798 (288); 241.05016 (469); 241.05234 (278); 241.05452 (136); 241.05669 (78); 241.05887 (96); 269.03961 (108); 269.04191 (167); 269.04421 (371); 269.04651 (351); 269.04881 (141); 269.05112 (166); 269.05342 (86); 298.04252 (87); 298.04495 (235);

											298.04737 (200); 298.04979 (250); 298.05221 (129); 298.05464 (90); 298.05706 (107)
66	Pachypodol	15.48	343.082	343.080	6.79	343.082	$C_{18}H_{16}O_7$	59.7	72.7	77.4	285.03289 (221); 285.03526 (200); 285.03763 (368); 285.04 (257); 285.04237 (400); 313.02621 (141); 313.0287 (306); 313.03118 (777); 313.03366 (588); 313.03614 (600); 313.03863 (495); 313.04111 (228); 328.05184 (160); 328.05693 (206); 328.05947 (162)
67	Pomiferin	17.74	419.150	419.151	3.31	419.150	$C_{25}H_{24}O_6$	20.7	100	67.7	115.91961 (309); 115.92112 (223); 419.14506 (425); 419.14794 (325); 419.15081 (591); 419.15368 (505); 419.15656 (424); 419.15943 (277)
68	3-Hydroxy-7,8,4'-trimethoxyflavone	18.05	327.087	327.083	14.41	327.087	$C_{18}H_{16}O_6$	23	57.1	63.6	125.0974 (12); 125.10054 (12); 165.09134 (12); 165.09315 (12); 169.02534 (12); 169.06001 (12); 171.09689 (12); 171.10423 (12); 171.13544 (12); 183.13157 (12); 183.14487 (12); 259.19879 (12); 259.20105 (12); 259.21009 (12); 265.17239 (12); 265.21809 (12); 265.22038 (12); 269.02531 (12); 269.04372 (24); 269.04603 (12); 270.08974 (12); 270.1151 (12); 281.15068 (12); 281.15303 (12); 281.15774 (12); 283.17788 (12); 283.21094 (12); 283.22983 (12); 297.006 (12); 297.02293 (12);

	297.02535 (12); 297.03018 (12); 297.0326 (12); 297.03502 (24); 297.03744 (36); 297.03986 (12); 297.04228 (12); 297.0447 (12); 297.04711 (24); 297.05437 (12); 312.058 (12); 312.06048 (12); 312.06296 (12); 312.06544 (12); 327.21292 (12); 327.21546 (12); 327.22054 (12); 327.22815 (12); 327.24592 (12); 327.251 (36)
